# An update on the prevalence of *Echinococcus multilocularis* in red foxes (*Vulpes vulpes*) in a Central European focus: Northern and northeastern Poland (2022–2024)

**DOI:** 10.1016/j.onehlt.2025.101151

**Published:** 2025-07-25

**Authors:** Paweł Gładysz, Agnieszka Świątalska, Małgorzata Samorek-Pieróg, Jacek Karamon, Anna Lass

**Affiliations:** a Division of Tropical Parasitology, Department of Tropical Medicine and Parasitology, Institute of Maritime and Tropical Medicine, Medical University of Gdańsk, Powstania Styczniowego 9B, 81-519 Gdynia, Poland; b Department of Veterinary Hygiene, Kaprów 10, 80-316 Gdańsk, Poland; c National Veterinary Research Institute, Partyzantów 57, 24-100 Puławy, Poland

**Keywords:** Alveolar echinococcosis, Zoonosis, Public health surveillance, Copro-PCR, 12S rDNA

## Abstract

Knowing *Echinococcus multilocularis* prevalence in wild canids helps us assess the risk of zoonotic spillover. In this study, we updated the infection statistics among red foxes in northern and northeastern Poland since the last monitoring effort at the district (Polish: *powiat*) level (2001–2004). We collected faecal samples from 192 red foxes from the districts of Słupsk, Puck, Wejherowo, Kościerzyna, and Kartuzy (Pomorskie Voivodship); 200 individuals from the districts of Bartoszyce, Kętrzyn, Gołdap, Iława, and Węgorzewo (Warmińsko-Mazurskie Voivodship); and 47 individuals from the district of Augustów (Podlaskie Voivodship). Based on nested PCR testing confirmed by Sanger sequencing, we calculated the prevalence with a 95 % confidence interval and compared the results to previous reports. We recorded the highest proportion of infected red foxes in Gołdap District (21/40, 52.5 %, 95 % CI: 37.5 %–67.1 %). The shared prevalence in Puck, Wejherowo, Kościerzyna, and Kartuzy Districts was 10.4 % (19/182, 95 % CI: 6.7 %–15.8 %). One red fox tested positive in Iława District (1/48, 2.1 %, 95 % CI: −0.6 %–11.9 %). We found no statistically significant changes in prevalence in the sampled areas. Our results indicate that *E. multilocularis* prevalence has remained stable in red fox populations throughout the region over the past 20 years.

## 1 Introduction

*Echinococcus multilocularis* Leuckart, 1863, is a small taeniid tapeworm of medical and One Health importance. Its proliferating larval stage causes damage to the liver of intermediate hosts and can metastasise to other organs [1]. Infected people develop alveolar echinococcosis (AE), a disease characterised by a long incubation period of up to several years and a high mortality rate of 90 % and higher without proper treatment [2]. AE is limited to the Northern Hemisphere, including areas of high endemicity in Qinghai–Tibet Plateau, China, Central Europe, and parts of Siberia [2]. The World Health Organization (WHO) acknowledges AE as a neglected tropical disease and advocates the implementation of control measures [3]. In 2022, a total of 2802 cases of echinococcosis (alveolar and cystic) were reported from 31 countries.

In Poland, the incidence of AE is hard to estimate because no distinction is made between alveolar and cystic echinococcosis in the national records, and there are reasons to believe that some AE cases are unreported (non-diagnosed or misdiagnosed). The latest and most complete data can be found in a paper by Nahorski et al. [4]; the analysis of all autochthonous human AE cases recorded in Poland from 1990 to 2011 indicated that Warmińsko-Mazurskie was the voivodship with the highest number of AE cases (53.7 %), with the average detection rate of human AE of 0.2 per 100,000 inhabitants, as compared with 0.014 per 100,000 inhabitants nationally. Since this publication, data on the disease are fragmentary and rather come from internal records of individual hospitals treating AE patients. For example, only at the University Centre for Maritime and Tropical Medicine, Medical University of Gdańsk (UCMTM)—one of the few specialised clinical centres in Poland where AE patients are treated—110 AE cases were diagnosed since 2000. Between 2017 and 2024, 38 patients received treatment due to AE, 16 of them from Warmińsko-Mazurskie Voivodship and eight from Pomorskie Voivodship (clinical data).

But humans are not the only species threatened by *E. multilocularis*. A limited number of studies highlight the risk of contracting AE by household animals. Infections in pigs were noted in northeastern and southeastern voivodships, indicating that pig farms can also be affected [5,6]. The risk of AE also extends to dogs—which are primarily regarded as definitive hosts [7]—with two cases of AE in pets from Małopolskie and Pomorskie Voivodships reported recently [8]. Research on the role of dogs, a potential source of infection for people, in *E. multilocularis* transmission in Poland is pending.

*Echinococcus multilocularis* is transmitted mainly in the sylvatic cycle, in which wild carnivores serve as definitive hosts, and many species of small rodents act as intermediate hosts [9]. Spillover to accidental intermediate hosts, including humans, results from environmental contamination with parasite eggs by the key definitive host. In Europe, it is the red fox (*Vulpes vulpes* (Linnaeus, 1758)) [10,11]. Contact with contaminated soil as well as consumption of contaminated raw fruit, vegetables or water may therefore lead to infection in humans [12]. Europeans likely become infected by consuming contaminated berries, vegetables, and mushrooms. It was shown that the majority of Polish AE patients live in rural areas and have a long-standing, often occupational, association with woodland [4], which suggests that close contact with the contaminated environment is an important risk factor.

In Poland, in the years 2012–2018, an attempt was made to study the environment in northern and northeastern Poland for the presence of *E. multilocularis* eggs. Szostakowska et al. [13] detected DNA of this tapeworm in a farmyard in Węgorzewo District and kitchen gardens in Iława District by collecting soil samples from selected areas frequented by red foxes in Warmińsko-Mazurskie Voivodship, providing proof of environmental contamination in people's immediate surroundings. Further studies in the voivodship demonstrated that forest berries, mushrooms, vegetables from kitchen gardens, and raspberries from small local plantations may be direct sources of human infection if gathered in places frequented by foxes (sampling sites were selected based on advice from locals familiar with fox behaviour) [14]. In Pomorskie Voivodship, parasite DNA was also detected in forest mushrooms and kitchen-garden vegetables but in a much smaller number of samples [15]. Moreover, three water samples collected in Warmińsko-Mazurskie Voivodship also contained *E. multilocularis* eggs, likely due to egg dispersal in surface run-off [16]. Though egg viability and pathogenic potential were not assessed, this finding substantiated another possible source of infection, albeit less relevant than contaminated food. Widespread presence of tapeworm eggs on plants, in soil, and in water can be traced back to infected definitive hosts in the area.

### 1.1 Past studies of *E. multilocularis* prevalence in Polish red foxes

*Echinococcus multilocularis* was first reported in Poland in the mid-1990s, based on its detection in two out of 20 red foxes shot near Gdańsk [17]. This discovery linked the Central European endemic area with the large endemic zone in Russia, indicating a uniform distribution.

In one of the two studies spanning 1994–1997, researchers inspected small-intestine mucosal smears from 1042 red foxes across 33 voivodships (pre-1999 administrative division), including northeastern Poland [18]. They identified tapeworms based on morphological features and detected *E. multilocularis* in 53 red foxes (5.1 %). The proportion of infected animals was highest in northeastern Poland (10.1 %), reaching up to 35.7 % in Suwalskie Voivodship. In a microscopic examination of 1909 red fox intestines collected during the same period in 15 voivodships of northwestern Poland, three out of 80 individuals (3.8 %) tested positive for *E. multilocularis* [19].

A regional screening for the years 2001–2004 involved 1514 red foxes from Pomorskie, Warmińsko-Mazurskie, and Podkarpackie Voivodships. Investigators employed a modified intestinal scraping technique (IST) followed by morphological identification [20]. They recorded the highest prevalence in Warmińsko-Mazurskie Voivodship (39.6 %), with the highest local prevalence in the districts of Gołdap and Olecko (62.9 %), and Kętrzyn (55.9 %). In Pomorskie Voivodship, 7.9 % of red foxes were positive for *E. multilocularis*. Most infected animals were found in the districts of Kartuzy (15.4 %) and Puck (15.0 %). In the 1990s, 9.5 % of red foxes in Podlaskie Voivodship harboured the tapeworm [21].

The monitoring report published in 2014 covered 15 out of 16 voivodships [22]. The sedimentation and counting technique (SCT) was used to inspect 1546 intestinal samples from red foxes shot between 2009 and 2013. Half of the Warmińsko-Mazurskie Voivodship foxes tested positive (95 % CI: 40.3 %–59.7 %). In Podlaskie Voivodship, about one-third of the fox population was infected (95 % CI: 25.5 %–43.7 %). There was a clear pattern in the distribution of positive animals: areas with higher prevalence (17.5 %–50.0 %) were in the east, while the western part was less affected (0.0 %–11.8 %). This division may result from the Vistula River limiting red fox movement [23]. The authors noted that since the late 1990s and early 2000s, prevalence increased in the east but not in the west. Interestingly, in the years 2009–2013, the prevalence in Pomorskie Voivodship was 3.0 % (95 % CI: 1.0 %–8.5 %), but an SCT inspection of Eastern Pomeranian red foxes hunted between September 2018 and October 2022 revealed that 16.5 % (95 % CI: 10.3 %–25.2 %) of individuals were infected with *E. multilocularis* [24]. The sampling area included the entire Pomorskie Voivodship, the area adjacent to the Vistula Lagoon, and a part of Warmińsko-Mazurskie Voivodship.

Prevalence in the adjacent territories of Kaliningrad Oblast (Russia) and Belarus has not been reported. Between 2001 and 2006 in Lithuania, 58.7 % of red foxes (95 % CI: 52.6 %–64.7 %) and 8.2 % of raccoon dogs (*Nyctereutes procyonoides* (Gray, 1834)) (95 % CI: 3.4 %–16.2 %) harboured *E. multilocularis* [25]. Two-way canid migrations through Suwałki and Sejny Districts in Podlaskie Voivodship, conducive to the parasite's dispersal, are possible. Positive immigrant foxes may maintain regional prevalence, while positive emigrant animals may contribute to prevalence in adjacent territories abroad.

As noted by Gawor and Borecka [26], a high number of AE cases in a limited area combined with a large population of infected definitive hosts suggests that there is a risk to humans due to a high level of environmental contamination with tapeworm eggs and the fact that local communities have a regular contact with the environment. Strategies for preventing human infections should be based on a holistic approach, consistent with the One Health concept, and include regular monitoring of definitive hosts, especially the red fox, combined with testing of environmental samples for the presence of *E. multilocularis* eggs.

### 1.2 Aim

We aimed to calculate the current prevalence of *E. multilocularis* in red foxes in selected districts of northern and northeastern Poland using copro-PCR, the most species-specific method for screening intestinal matter [27,28].

## 2 Materials and methods

### 2.1 Sampling

We conducted the study in 11 districts across Pomorskie, Warmińsko-Mazurskie, and Podlaskie Voivodships selected based on AE incidence [4] and the logistics of material collection. The project received approval from the Medical University of Gdańsk Bioethics Committee for Scientific Research (decision no. NKBBN/467/2021). Red foxes and raccoon dogs from each location were collected in collaboration with the Polish Hunting Association and the General Directorate of State Forests and transported to the Institute of Veterinary Hygiene in Gdańsk for necropsy. The removed intestines were then sent to the National Veterinary Research Institute in Puławy for content collection.

**Fig. 1:**
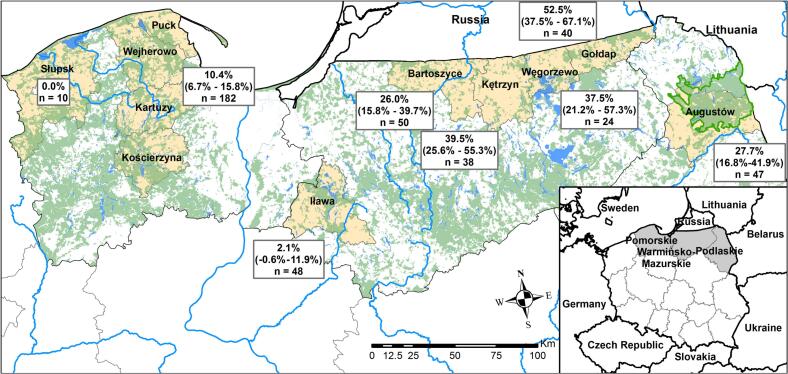
Map of the study area showing sampled districts (yellow), forests (green), and major lakes and rivers (blue). The thick green line delineates the perimeter of the Augustów Primeval Forest. The prevalence of *Echinococcus multilocularis* in red foxes is presented with corresponding 95 % confidence intervals (CIs) and sample sizes (n). (For interpretation of the references to colour in this figure legend, the reader is referred to the web version of this article.)

Hunters from Pomorskie Voivodship provided us with 182 red foxes and three raccoon dogs from the districts of Kartuzy, Kościerzyna, Wejherowo, and Puck during two ceremonial displays of game killed on hunts organised by local hunting clubs in January and February 2022. We selected the animals based on the degree of injury; individuals with visible abdominal wounds indicative of digestive tract lacerations were discarded. Each carcass was assigned a number and placed in a separate plastic bag for sanitary transportation. Several hunting clubs participated in these events. Puck, Wejherowo, Kościerzyna, and Kartuzy Districts were treated as one sampling area because hunters piled foxes from different hunting zones. Additionally, we received ten red foxes from Słupsk District in January 2023 (Fig. 1).

Animals in Warmińsko-Mazurskie Voivodship were collected throughout 2023 and 2024. We obtained 50 red foxes and three raccoon dogs from Bartoszyce District, 38 red foxes from Kętrzyn District, 24 red foxes from Węgorzewo District, 40 red foxes and one raccoon dog from Gołdap District (all four areas border Kaliningrad Oblast), and 48 red foxes from Iława District (just outside Pomorskie Voivodship)—totalling 200 red foxes and four raccoon dogs. In the first half of 2023, a hunting club in Augustów provided us with 47 red foxes and one raccoon dog from Augustów District, Podlaskie Voivodship, bordering Belarus (Fig. 1).

The age of adult animals was determined based on counting dentine layers on longitudinally sanded canine roots [29] and tooth attrition [30]. Red foxes aged one year and older were considered adults. Accounting for the limitations of age estimates provided by necropsy technicians, we divided the animals into four groups: [0,1), [1,2), [2,3], and over three years of age.

### 2.2 DNA extraction

The intestines were deep-frozen at a minimum of −70 °C for at least 7 days to kill oncospheres [28]. A 500-μl portion of faecal matter collected from the rectum, or contents of the posterior part of the small intestine, was then stored at −20 °C until DNA extraction using Genomic Mini AX Stool (A&A Biotechnology, cat. no. 065–60, Gdańsk, Poland), a kit with increased efficiency for genomic DNA purification from faeces (procedure with DNA precipitation). Prior to step 1 of the manufacturer's protocol, samples were subjected to three freeze-thaw cycles to facilitate the release of soft tissue from eggs: intestinal contents mixed with 750 μl of lysis buffer were kept at −70 °C for 30 min (until completely frozen), then moved to 65 °C for 5 min. The DNA pellet was dissolved in 100 μl of Tris buffer (10 mM Tris-HCl, pH 8.5).

### 2.3 Detection limit

To measure the sensitivity of our diagnostic method, we performed the nested PCR protocol outlined below in triplicate, using an artificial construct—a plasmid pJET1 with an insert containing hybridisation sites for external and internal primers (pJET1_Em)—in serial dilutions from 10^10^ to 10^0^ copies per microlitre.

### 2.4 Nested PCR and sequencing

*First PCR.* The total reaction volume of 25 μl contained 12.5 μl of PCR Mix Plus Green (A&A Biotechnology, cat. no. 2005-1000Z, Gdańsk, Poland), forward primer p60for (5′–TTAAGATATATGTGGTACAGGATTAGATACCC–3′) and reverse primer p375rev (5′–AACCGAGGGTGACGGGCGGTGTGTACC–3′)—a pair specific for the 12S rDNA sequence of Taeniidae [31]—each added to a final concentration of 0.4 μM, 8.5 μl of sterile, nuclease-free, DEPC-treated water, and 2 μl of a DNA extract. Each batch of samples was tested alongside a negative control (2 μl of sterile, nuclease-free, DEPC-treated water) and a positive control (2 μl of a DNA extract from an adult tapeworm). The expected product size was 373 bp. PCR conditions were as follows: 95 °C for 5 min; 50 cycles of 95 °C for 10 s, 55 °C for 30 s, and 72 °C for 70 s; with a final elongation step at 72 °C for 7 min.

*Second PCR.* The total reaction volume of 25 μl contained 12.5 μl of PCR Mix Plus Green, forward primer Em-nest-for (5′–GTGAGTGATTCTTGTTAGGGGAAGA–3′) and reverse primer Em-nest-rev (5′–ACAATACCATATTACAACAATATTCCTATC–3′)—a pair specific for *E. multilocularis* [32]—each added to a final concentration of 0.4 μM, 9.5 μl of sterile, nuclease-free, DEPC-treated water, and 1 μl of a 10-times diluted product of the first PCR. The expected product size was 204 bp. PCR conditions were as follows: 95 °C for 15 min; 35 cycles of 95 °C for 40 s, 60 °C for 40 s, and 72 °C for 1 min; with a final elongation step at 72 °C for 7 min.

Products of the second reaction were visualised on a 2 % agarose gel. Positive samples were reamplified in a total reaction volume of 20 μl containing 10 μl of Platinum™ SuperFi II Green PCR Master Mix (Thermo Fisher Scientific™, cat. no. 12369010), modified forward primer Em-nest-M13F (5′–**GTAAAACGACGGCCAG**GTGAGTGATTCTTGTTAGGGGAAGA–3′) and modified reverse primer Em-nest-M13R (5′–**CAGGAAACAGCTATGAC**ACAATACCATATTACAACAATATTCCTATC–3′), each added to a final concentration of 0.4 μM, 7 μl of sterile, nuclease-free, DEPC-treated water, and 1 μl of a 10-times diluted product of the first PCR. The underlined primer sections are hybridisation sites for standard sequencing primers M13F and M13R. PCR conditions were as follows: 98 °C for 30 s; 35 cycles of 98 °C for 10 s, 60 °C for 10 s, and 72 °C for 30 s; with a final elongation step at 72 °C for 5 min.

Bidirectional sequencing of final amplicons was outsourced to Macrogen Europe (Amsterdam). Raw forward and reverse reads were trimmed and assembled in Geneious Prime (https://www.geneious.com/prime/, GraphPad Software, LLC (d.b.a. Geneious), Boston, MA, USA). Species identification was confirmed by querying BLAST with consensus sequences (https://blast.ncbi.nlm.nih.gov/Blast.cgi).

### 2.5 Inhibition control

Genetic material extracted from intestinal contents and faecal matter may contain contaminants, such as bile salts or complex polysaccharides [33,34]. We tested all negative samples for the presence of polymerase inhibitors by performing PCRs spiked with pJET1_Em, according to the protocol for the secondary PCR outlined above. A total reaction mixture of 25 μl contained 12.5 μl of PCR Mix Plus Green, each primer added to a final concentration of 0.4 μM, 7.5 μl of sterile, nuclease-free, DEPC-treated water, 1 μl of pJET1_Em (10^8^ copies per microlitre), and 2 μl of a DNA extract. PCR products were visualised on a 2 % agarose gel.

### 2.6 Statistical analysis

We estimated the 95 % confidence intervals (95 % CI) for proportions of infected red foxes using the package ‘binom’ [35] in R version 4.2.3 [36], following the guidelines in [37]. For sampling sizes of *n* ≤ 40, we implemented the Wilson interval. For sampling sizes of *n* > 40, we used the Agresti-Coull interval.

The obtained proportions were compared with previous reports by performing Fisher's exact tests for count data (α = 0.05) in STATISTICA Zestaw Plus version 5.0.96 (https://www.statsoft.pl/, StatSoft Polska Sp. z o. o., Kraków, Poland). Using Pearson's chi-squared test, we verified the null hypothesis of no association between age group or sex and the number of positive samples in two groups: the north (Pomorskie Voivodship) and northeast (Warmińsko-Mazurskie Voivodship and Augustów District, Podlaskie Voivodship) (α = 0.05). Samples of unknown sex and/or age were excluded.

## 3 Results

### 3.1 Detection limit

**Supplementary Fig. 1:**
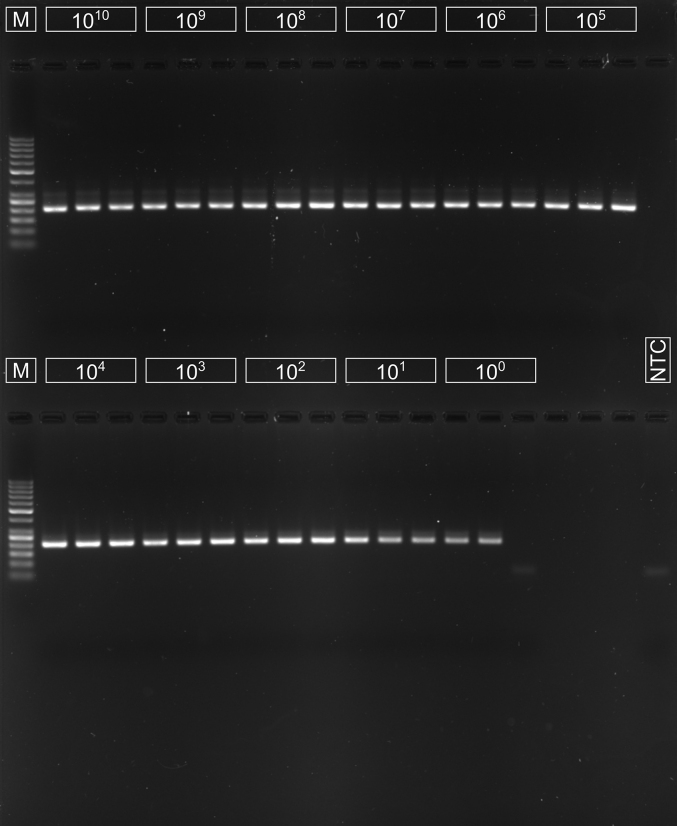
Nested PCR sensitivity test: products of the secondary reaction visualised on a 2 % agarose gel. M – GeneRuler 50 bp DNA Ladder (Thermo Scientific™, cat. no. SM0371); NTC – no-template control.

The sensitivity of the nested PCR assay was one copy of the template per microlitre (Supplementary Fig. 1).

### 3.2 Nested PCR and sequencing

Nineteen out of 182 samples (10.4 %) tested positive in Puck, Wejherowo, Kartuzy, and Kościerzyna Districts. We found no positive individuals in Słupsk District. Fifty-nine out of 200 samples (29.5 %) from Warmińsko-Mazurskie Voivodship and thirteen out of 47 extracts from Augustów District (27.7 %) contained *E. multilocularis* DNA. All eight raccoon dogs tested negative (Supplementary File 1). Sequencing was successful for all amplicons and confirmed species identification. We deposited the obtained 12S rDNA sequence fragments (204 bp) in GenBank (accessions PQ199857–PQ199947).

Sample G-295 from Pomorskie Voivodship gave a faint band in agarose gel electrophoresis, so we reamplified and sequenced the product. The resulting 12S rDNA sequence belonged to *Echinococcus intermedius* (G7) (Per. Ident. = 97.06 %, *E*-value = 3e-55, Accession: MT396434.1).

A detailed sample list is available in Supplementary File 2.

### 3.3 Inhibition control

Samples G-302, G-303, and G-501 showed visibly weaker bands in agarose gel electrophoresis. These DNA extracts were diluted tenfold, and nested PCR was repeated for dilutions spiked with pJET1_Em and clean. All three extracts tested negative. We detected no false-negative samples (Supplementary File 3).

### 3.4 Statistical analysis

**Fig. 2:**
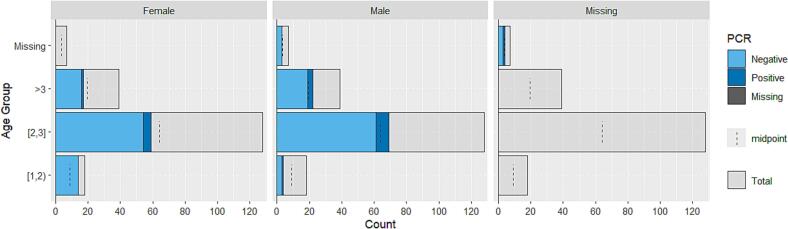
Population pyramid for the districts of Pomorskie Voivodship. The number of red foxes is shown by age group, sex, and infection status. (For interpretation of the references to colour in this figure legend, the reader is referred to the web version of this article.)

**Fig. 3:**
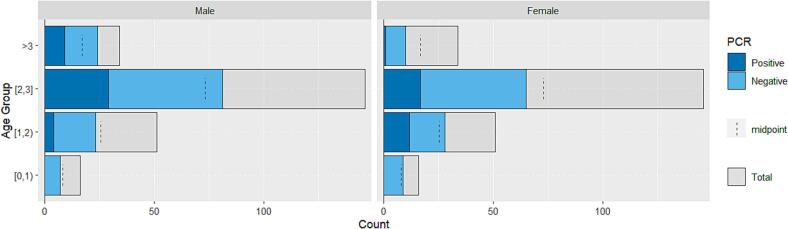
Population pyramid for the districts of Warmińsko-Mazurskie Voivodship and Augustów District, Podlaskie Voivodship. The number of red foxes is shown by age group, sex, and infection status. (For interpretation of the references to colour in this figure legend, the reader is referred to the web version of this article.)

Most red foxes in the north and northeast were aged 2–3 years (Fig. 2, Fig. 3). The only juveniles tested came from the districts of Iława (9 individuals), Augustów (5 individuals), and Węgorzewo (2 individuals), all of which tested negative. Age was unknown for seven red foxes from Pomorskie Voivodship, four of which also lacked information about sex. One of these tested positive. Prevalence was highest in Gołdap District, with 21 out of 40 red foxes infected (52.5 %, 95 % CI: 37.5 %–67.1 %) (Table 1). One red fox tested positive in Iława District (2.1 %, 95 % CI: −0.6 %–11.9 %). We found no significant association between age group or sex and the number of positive samples in the north (Χ^2^_Age [df = 2, *N* = 185] = 0.40, p_Age = 0.82; Χ^2^_Sex [df = 1, *N* = 188] = 1.69, p_Sex = 0.19) or in the northeast (Χ^2^_Age [df = 3, *N* = 247] = 7.10, p_Age = 0.07; Χ^2^_Sex [df = 1, N = 247] = 0.55, p_Sex = 0.46). The results of the two-tailed Fisher's exact tests comparing historical and current data are shown in Table 1.

**Table 1:** Results of two-tailed Fisher's exact tests for count data (α = 0.05) comparing current and historical prevalence of *Echinococcus multilocularis* among red foxes in relevant areas.

2 × 2 Contingency Table No.	Studied Area	Examined	Positive	Prevalence [%]	95 % CI [%]	Sampling Date	Source	Reference Area	Examined	Positive	Prevalence [%]	95 % CI [%]	Sampling Date	Source	p
1	Słupsk District	10	0	0.0	0.0–27.8	Jan 2023	This study	Słupsk District	12	1	8.3	1.5–35.4	2001–2004	[20]	1.0000
2	Puck, Wejherowo, Kartuzy, Kościerzyna Districts	182	19	10.4	6.7–15.8	Jan/Feb 2022	This study	Puck, Wejherowo, Kartuzy, Kościerzyna Districts	196	18	9.2	5.8–14.1	2001–2004	[20]	0.7309
3	Puck, Wejherowo, Kartuzy, Kościerzyna Districts	182	19	10.4	6.7–15.8	Jan/Feb 2022	This study	Pomorskie Voivodship	100	3	3.0	0.7–8.8	2009–2013	[22]	0.0346
4	Puck, Wejherowo, Kartuzy, Kościerzyna Districts	182	19	10.4	6.7–15.8	Jan/Feb 2022	This study	Eastern Pomerania	97	16	16.5	10.3–25.2	Sep 2018 – Oct 2022	[24]	0.1835
5	Bartoszyce	50	13	26.0	15.8–39.7	2023–2024	This study	Bartoszyce District	8	4	50.0	21.5–78.5	2001–2004	[20]	0.2157
6	Kętrzyn District	38	15	39.5	25.6–55.3	2023–2024	This study	Kętrzyn District	34	19	55.9	39.5–71.1	2001–2004	[20]	0.2371
7	Gołdap District	40	21	52.5	37.5–67.1	2023–2024	This study	Gołdap and Olecko Districts	35	22	62.9	46.3–76.8	2001–2004	[20]	0.4833
8	Iława District	48	1	2.1	−0.6-11.9	2023–2024	This study	Iława District	7	0	0.0	0.0–35.4	2001–2004	[20]	1.0000
9	Węgorzewo District	24	9	37.5	21.2–57.3	2023–2024	This study	Węgorzewo District	9	2	22.2	6.3–54.7	2001–2004	[20]	0.6808
10	Augustów District	47	13	27.7	16.8–41.9	2023	This study	Podlaskie Voivodship	100	34	34.0	25.4–43.7	2009–2013	[22]	0.5698
11	Augustów District	47	13	27.7	16.8–41.9	2023	This study	Augustów Primeval Forest	66	4	6.1	1.9–15.0	2014–2015	[42]	0.0026

For comparability, 95 % confidence intervals were calculated as in Materials and methods.

## 4 Discussion

Monitoring of *E. multilocularis* prevalence in red foxes—especially in endemic and potentially newly-endemic areas—is essential because these carnivores are the main spreaders of the parasite's eggs. The One Health link between red fox infections and the resulting environmental threat to humans is recognised internationally. Studies in recent years focused on such questions as ecological factors associated with fox faeces density in an *E. multilocularis* endemic area [38], assessing the role of individual foxes in environmental contamination with *E. multilocularis* through faecal samples [39], and testing whether the unequal distribution of human cases in an endemic focus is reflected in the small-scale distribution of *E. multilocularis* in foxes [40]. The first infected red fox in Bosnia and Herzegovina was captured in a place where immigrants from the Middle and Central East temporarily stay in improvised shelters on the bare ground—adding a new social perspective to the ongoing discussion on *E. multilocularis* threat [41]. Meanwhile, new data on environmental contamination with *E. multilocularis* eggs in northern and northeastern Poland and the epidemiological uncertainty regarding AE incidence warranted a more detailed study of local red fox populations and a threat they pose, especially to rural communities.

### 4.1 Prevalence changes over time

In Warmińsko-Mazurskie Voivodship, in the study spanning 2001–2004, none of the seven red foxes in Iława District tested positive [20]. Among the red foxes shot in 2023, we found one infected individual out of 48 tested. This fox, aged 1 year, could have migrated from an adjacent area. Twenty years ago, the prevalence in surrounding districts ranged from 10 % to 30 %. To explain the proportion observed today, the epidemiological status of Iława District should be examined by considering land cover and climatic conditions. In 2017, two AE cases from villages in Iława District were diagnosed at the UCMTM (clinical data), indicating that AE is definitely a threat for people living in this part of the voivodship.

We calculated a difference of 16.4 percentage points in the prevalence in Kętrzyn District between 2001 and 2004 and 2023–2024. The number of examined red foxes was similar in both studies (34 and 38, respectively). The decrease from 55.9 % to 39.5 % was not statistically significant. Similarly, we noted a 10.2 percentage-point decrease in the prevalence in Gołdap District compared to the previous study (Gołdap-Olecko Districts) and a 15.3 percentage-point increase in Węgorzewo District. These differences were not significant. Our results suggest no change in the extent of infection among red foxes over the past 20 years.

Historical data for Bartoszyce District are insufficient to draw conclusions about prevalence changes. Twenty years ago, researchers tested only eight red foxes. In our study, we detected infection in thirteen out of 50 animals. The decrease from 50.0 % to 26.6 % was not statistically significant given the sample sizes.

Located between Wigry National Park in the north and Biebrza National Park in the south, 46.4 % of Augustów District (as of 2022) is covered by woodland (https://dbw.stat.gov.pl/en/baza-danych). The Augustów Primeval Forest constitutes a major part of the local sylvatic ecosystem. The prevalence of 27.7 % in our sampling areas, mostly adjacent to the primeval forest, was significantly higher than the 6.1 % prevalence calculated based on the SCT study of 66 red foxes hunted between 2014 and 2015 within the Augustów Primeval Forest proper [42]. However, it did not significantly differ from the 34.0 % prevalence calculated a decade ago for Podlaskie Voivodship based on 100 animals [22]. This difference can be explained by vulpine predatory behaviour. Forests are where red foxes set up dens, breed, and rest during the day. They forage elsewhere because small-mammal density in sylvatic habitats is low [43].

When it comes to the neighbouring Pomorskie Voivodship, twenty years ago, one out of 12 red foxes from Słupsk District tested positive (8.3 %) [20]. We did not detect any infected individuals among our ten samples. Considering the small sampling sizes in both studies, Słupsk District requires a robust re-examination, especially considering that AE is diagnosed in this area (one AE case in 2024 at the UCMTM (clinical data)).

The shared prevalence in Puck, Wejherowo, Kościerzyna, and Kartuzy Districts was similar to the pooled prevalence of 9.2 % for the years 2001–2004 and supports calculations made for Eastern Pomerania in an overlapping sampling period from September 2018 to October 2022 [24]. However, it exceeds the value calculated for Pomorskie Voivodship [22]. Red foxes in these parts of the voivodship may pose a risk to rural communities engaged in growing vegetables or collecting mushrooms.

It seems that the last study at the voivodship level distorted the view of the epidemiological situation by averaging out smaller-scale prevalence [22]. The reported prevalence of 50.0 % (95 % CI: 40.3 %–59.7 %) in Warmińsko-Mazurskie Voivodship, based on the examination of 98 red foxes, has reinforced the perception of the entire administrative area as a hyperendemic region. Our study suggests that the distribution of infected red foxes is more nuanced, possibly due to barriers to fox migration, such as water bodies and highways.

Relying on rough, broad-scale calculations may negatively impact AE control and prevention by directing resources to hypoendemic areas (such as Iława District) instead of hyperendemic ones (such as Gołdap District). Accordingly, decision-making informed by the relatively low prevalence in Pomorskie Voivodship (3.0 %) may overlook the AE risk in the districts of Puck, Wejherowo, Kartuzy, and Kościerzyna (10.4 %). To further substantiate this view, a similar study should be conducted in the neighbouring districts.

### 4.2 Small-scale differences and land cover

Micro-scale habitat variation and the formation of transmission hotspots [43] could explain differences in the number of infected animals between districts within one ecologically heterogeneous endemic voivodship. *Echinococcus multilocularis* prevalence in red foxes depends on landscape composition [43,44]. In Poland, it showed a very weak positive correlation with forest cover and a very weak negative correlation with agricultural land [22]. These land cover categories, however, may have been too broad and did not account for red fox habitat selectivity [45].

As predators, red foxes prefer fragmented and diverse landscapes, which offer an abundance of favoured prey. Food availability shapes dispersal and, in turn, egg deposition. Red foxes cover up to over 10 km each night searching for sustenance [43]. Large areas of continuous forest and arable fields (except for about a 300-m edge zone)—where food resources are less predictable—and water bodies are costly to move through and significantly limit migration, as seen in the Masurian Lake District [45,46]. As a result, the meeting risk between definitive and intermediate hosts in forests and fields is low [47]. Interestingly, seven out of eight cases from Pomorskie Voivodship diagnosed at the UCMTM between 2017 and 2024 were from districts topographically connected to Warmińsko-Mazurskie Voivodship and separated from the rest of Pomorskie Voivodship by the Vistula River.

The probability of a suitable intermediate host ingesting viable eggs partly depends on the coincidence of egg and population density, the former being a function of red fox defecating behaviour. A spatial overlap between preferred red fox defecation sites and intermediate host distribution is observed. In the countryside, micro-foci of parasite transmission from red foxes to rodents and back form on the edges of medium-height vegetation (e.g., meadows or ploughed fields) and road banks, following spatial aggregation of prey and potentially contaminative material left by foraging carnivores [43,47]. In such hotspots, shade and humidity preserve eggs.

### 4.3 Raccoon dogs

All eight raccoon dogs tested negative for *E. multilocularis*. The biological competence of this invasive species to host mature tapeworms is comparable to that of foxes [48], but the raccoon dog remains underresearched in Poland [49]. It is known to participate in the *E. multilocularis* life cycle in Pomorskie Voivodship [50], where two out of 24 individuals—one from Starogard Gdański and one from Puck—tested positive, with low and medium worm burdens, respectively.

Research conducted in Lithuania [25] revealed that prevalence and intensity of *E. multilocularis* infections are higher in red foxes (58.7 % [95 % CI: 52.6 %–64.7 %], mean worm burden of 526 [95 % CI: 369–785]) than in raccoon dogs (8.2 % [95 % CI: 3.4 %–16.2 %], mean worm burden of 41 [95 % CI: 9–934]). Similar synchronic data for Poland are lacking. For a long time, the only estimate available was a prevalence of 10.4 % (95 % CI: 4.1 %–19.3 %) pooled by [49] from two regional studies [50,51] and the European Union summary report on trends and sources of zoonoses, zoonotic agents, and food-borne outbreaks in 2013 [52]. A recent study, limited to northern Poland, reported a prevalence of 10.42 % in 96 necropsied raccoon dogs obtained from September 2018 to October 2021 [53]. Conversely, an examination of 53 raccoon dogs from the Augustów Primeval Forest yielded no positive results [42].

We have yet to learn to what extent raccoon dogs shape *E. multilocularis* transmission, for instance, by competing with red foxes for intermediate hosts. Red foxes consume more small rodents than raccoon dogs (whose diet is more plant-based), especially in the coldest months, when raccoon dogs hibernate [54,55]. What also seems to make raccoon dogs less relevant for transmission intensity is their reduced capacity for egg dispersal due to defecation in latrines [56]. Genetic examination of small-predator faeces deposited in the environment can clarify the importance of particular definitive hosts for the *E. multilocularis* life cycle.

## 5 Conclusions

Molecular testing of intestinal contents revealed the current prevalence of *E. multilocularis* among red foxes at the district level. We did not find statistically significant changes in the sampled areas of northern and northeastern Poland. Although the 95 % confidence intervals in this and all previous studies were broad, the consistency of results over time and across locations supports the inference that *E. multilocularis* prevalence has remained stable in red fox populations throughout the region over the past 20 years. Consequently, Warmińsko-Mazurskie remains a voivodship where the risk of contracting AE is among the highest in the country.

The following are the supplementary data related to this article.

Supplementary File 1 Products of the secondary nested PCR reaction visualised on 2 % agarose gels. Supplementary File 1

Supplementary File 2 Sample list with information on age, age group, sex, hunting area, source of material for DNA extraction, and nested PCR result for each red fox and raccoon dog. Supplementary File 2

Supplementary File 3 Inhibition control: PCR products visualised on 2 % agarose gels. Supplementary File 3

## Funding

This work was supported by the 10.13039/501100004281National Science Centre grant no. 2020/37/B/NZ7/03934.

## CRediT authorship contribution statement

**Paweł Gładysz:** Conceptualization, Methodology, Formal analysis, Investigation, Data curation, Writing – original draft, Writing – review & editing, Visualization. **Agnieszka Świątalska:** Resources, Writing – review & editing. **Małgorzata Samorek-Pieróg:** Resources. **Jacek Karamon:** Resources, Writing – review & editing. **Anna Lass:** Conceptualization, Methodology, Resources, Writing – review & editing, Supervision, Project administration, Funding acquisition.

## Declaration of competing interest

The authors declare no competing interests.

## Data Availability

The 12S rDNA sequence fragments obtained in this study (204 bp) have been deposited in GenBank under accession numbers PQ199857-PQ199947.
