# Supplementary material for: An update on the prevalence of Echinococcus multilocularis in red foxes (Vulpes vulpes) in a Central European focus: Northern and northeastern Poland (2022–2024)
Source: One Health. 2025 Jul 25;21:101151. doi: 10.1016/j.onehlt.2025.101151 (PMC12329506; doi:10.1016/j.onehlt.2025.101151)
Supplement: Supplementary File 1 — Products of the secondary nested PCR reaction visualised on 2 % agarose gels. [file mmc2.pdf]

NESTED PCR RESULTS

Products of the secondary nested PCR reaction visualised on 2% agarose gels. M – GeneRuler 50 bp DNA Ladder (Thermo Scientific, cat. no. SM0371); NTC – no-template control; PC – positive control.

AUGUSTÓW DISTRICT, PODLASKIE VOIVODSHIP

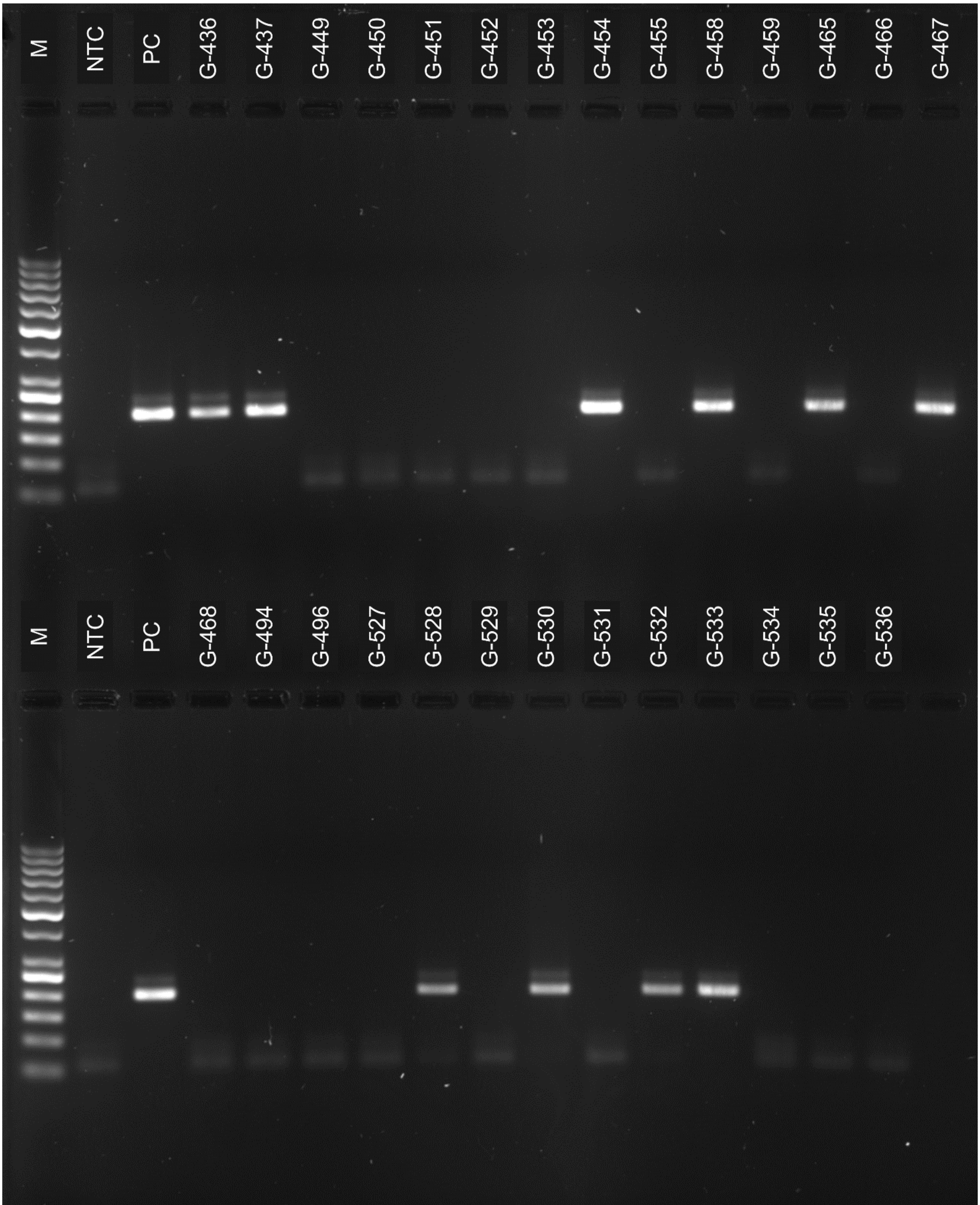

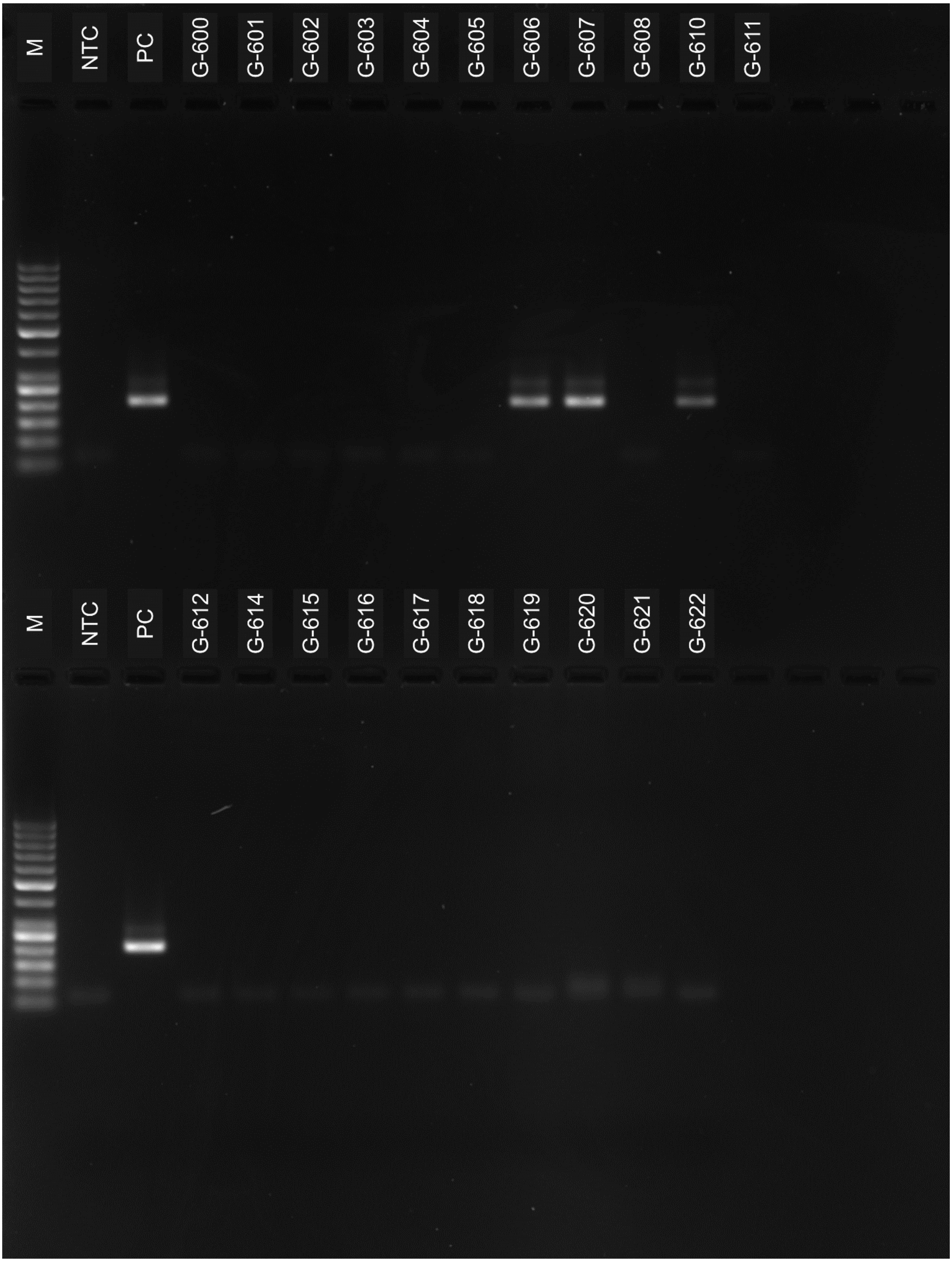
M
NTC
PC
G-600
G-601
G-602
G-603
G-604
G-605
G-606
G-607
G-608
G-610
G-611
M
NTC
PC
G-612
G-614
G-615
G-616
G-617
G-618
G-619
G-620
G-621
G-622

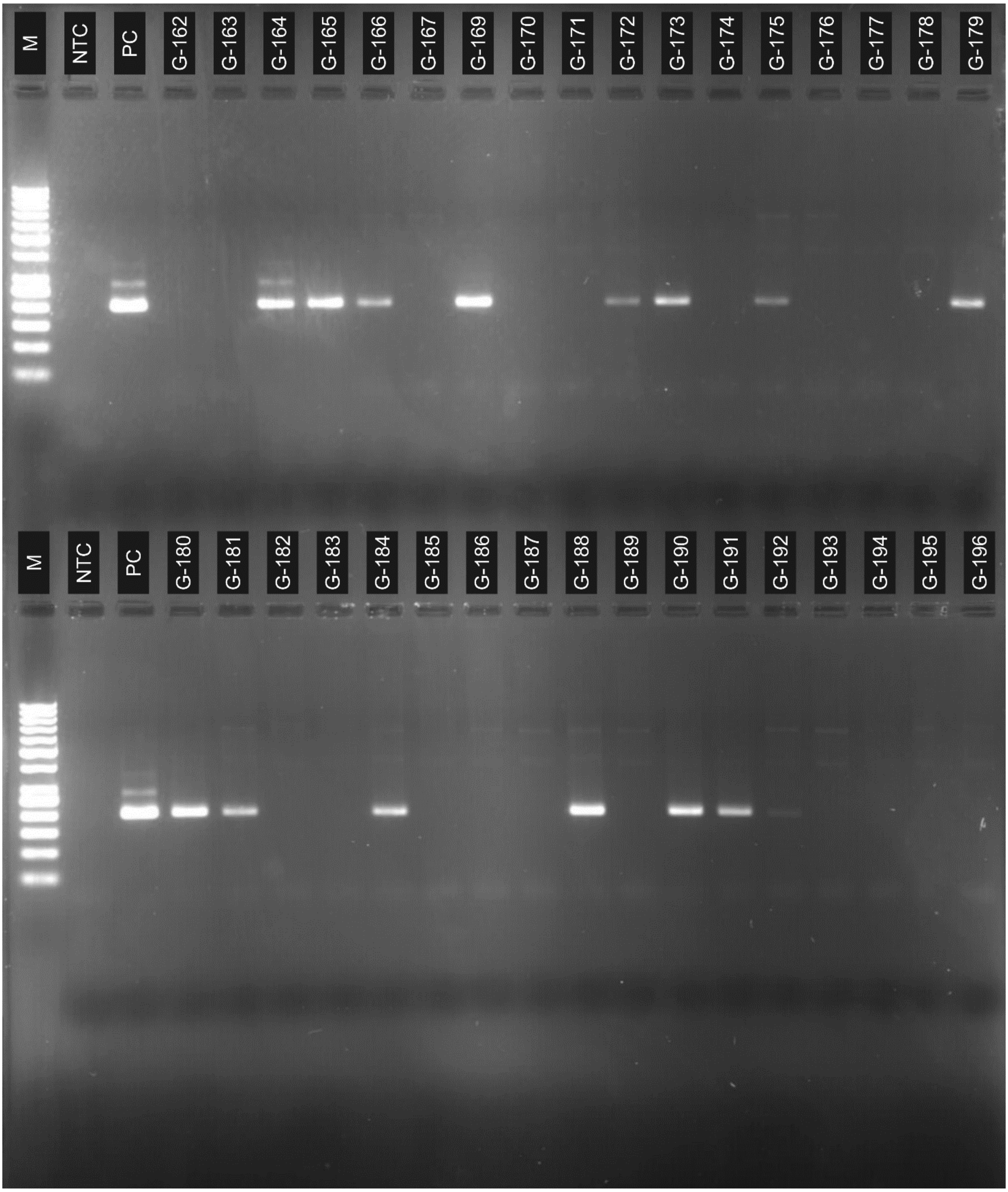
M
NTC
PC
G-162
G-163
G-164
G-165
G-166
G-167
G-169
G-170
G-171
G-172
G-173
G-174
G-175
G-176
G-177
G-178
G-179
M
NTC
PC
G-180
G-181
G-182
G-183
G-184
G-185
G-186
G-187
G-188
G-189
G-190
G-191
G-192
G-193
G-194
G-195
G-196

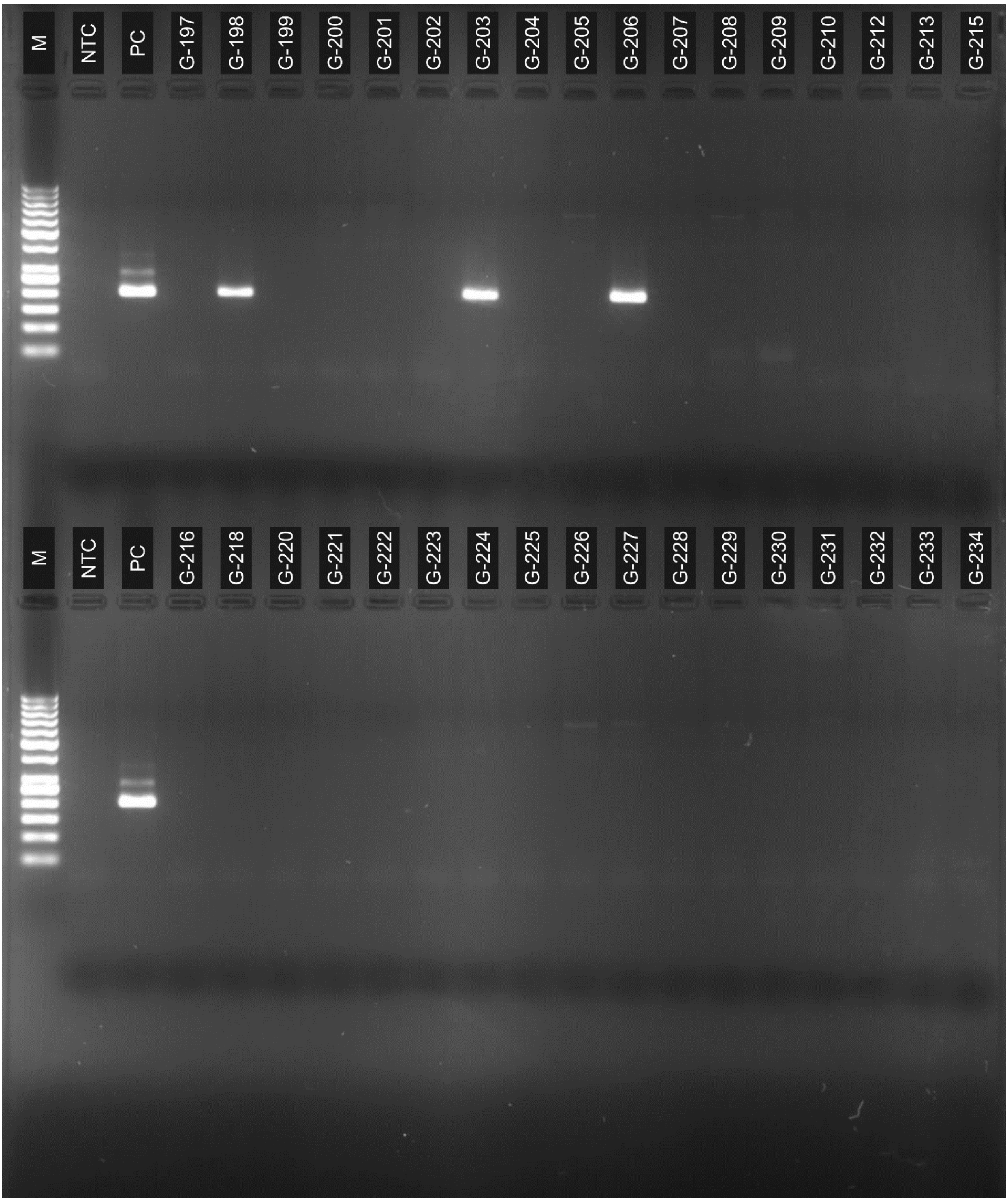
M
NTC
PC
G-197
G-198
G-199
G-200
G-201
G-202
G-203
G-204
G-205
G-206
G-207
G-208
G-209
G-210
G-212
G-213
G-215
M
NTC
PC
G-216
G-218
G-220
G-221
G-222
G-223
G-224
G-225
G-226
G-227
G-228
G-229
G-230
G-231
G-232
G-233
G-234

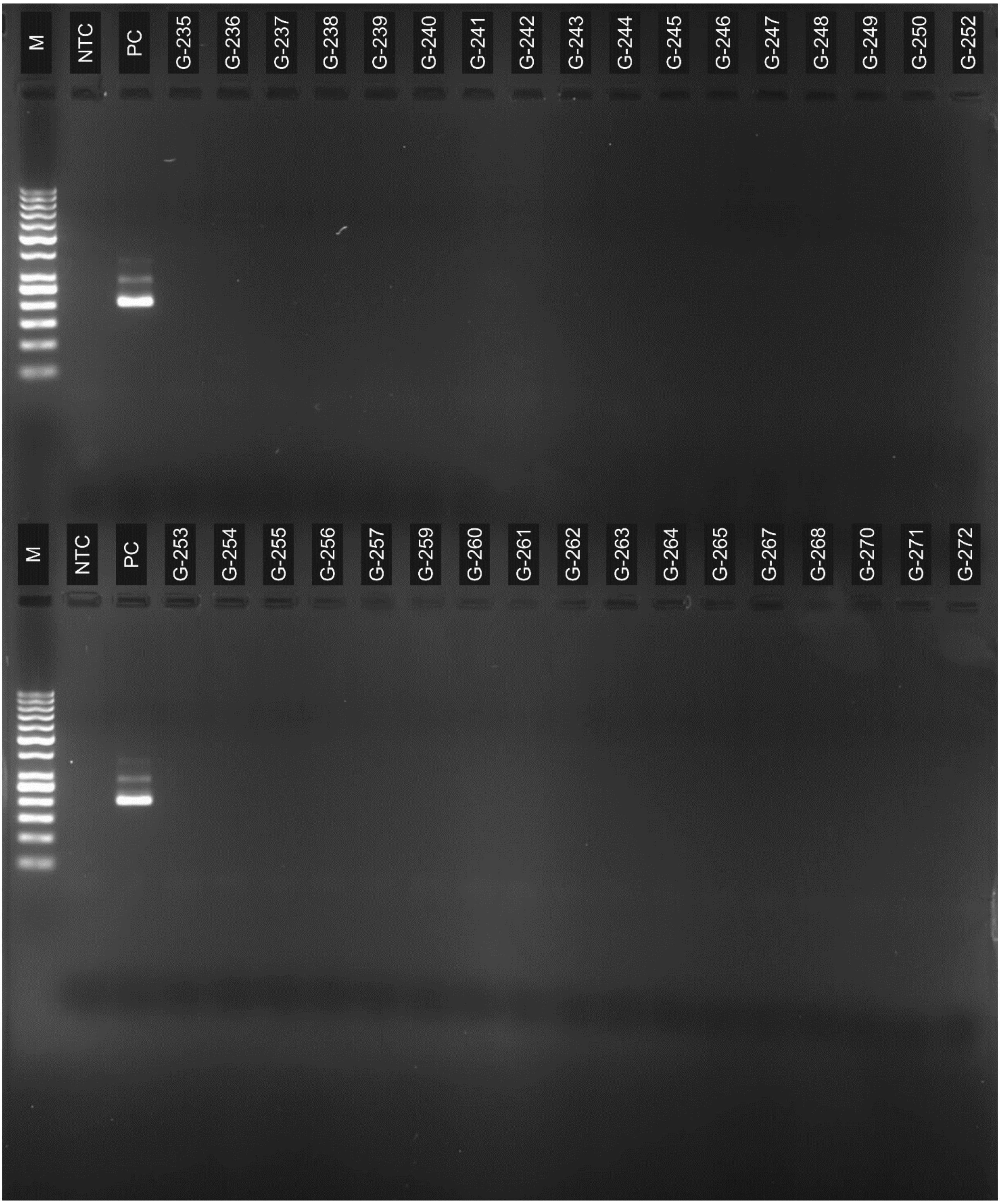
M
NTC
PC
G-235
G-236
G-237
G-238
G-239
G-240
G-241
G-242
G-243
G-244
G-245
G-246
G-247
G-248
G-249
G-250
G-252
M
NTC
PC
G-253
G-254
G-255
G-256
G-257
G-259
G-260
G-261
G-262
G-263
G-264
G-265
G-267
G-268
G-270
G-271
G-272

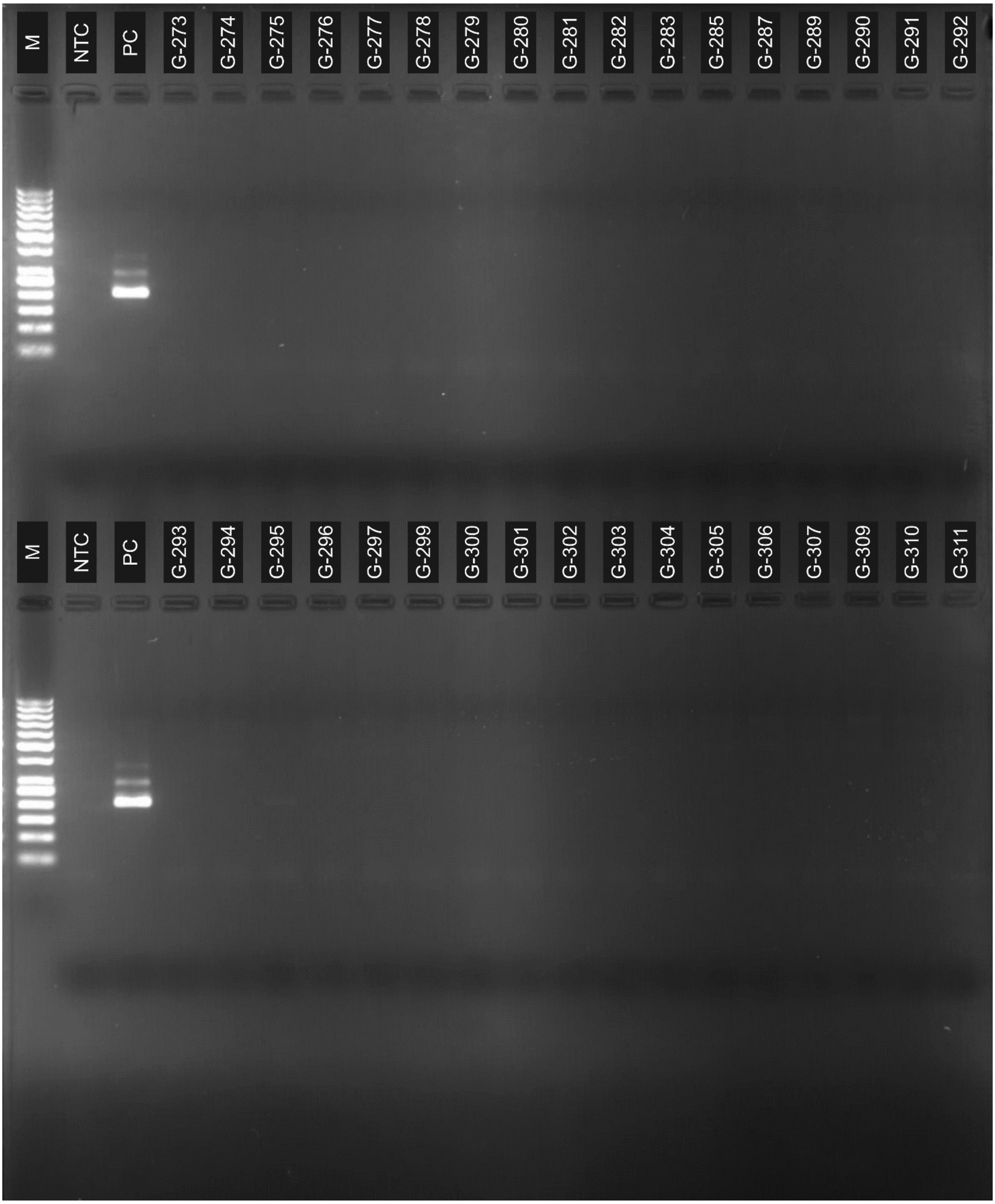
M
NTC
PC
G-273
G-274
G-275
G-276
G-277
G-278
G-279
G-280
G-281
G-282
G-283
G-285
G-287
G-289
G-290
G-291
G-292
M
NTC
PC
G-293
G-294
G-295
G-296
G-297
G-299
G-300
G-301
G-302
G-303
G-304
G-305
G-306
G-307
G-309
G-310
G-311

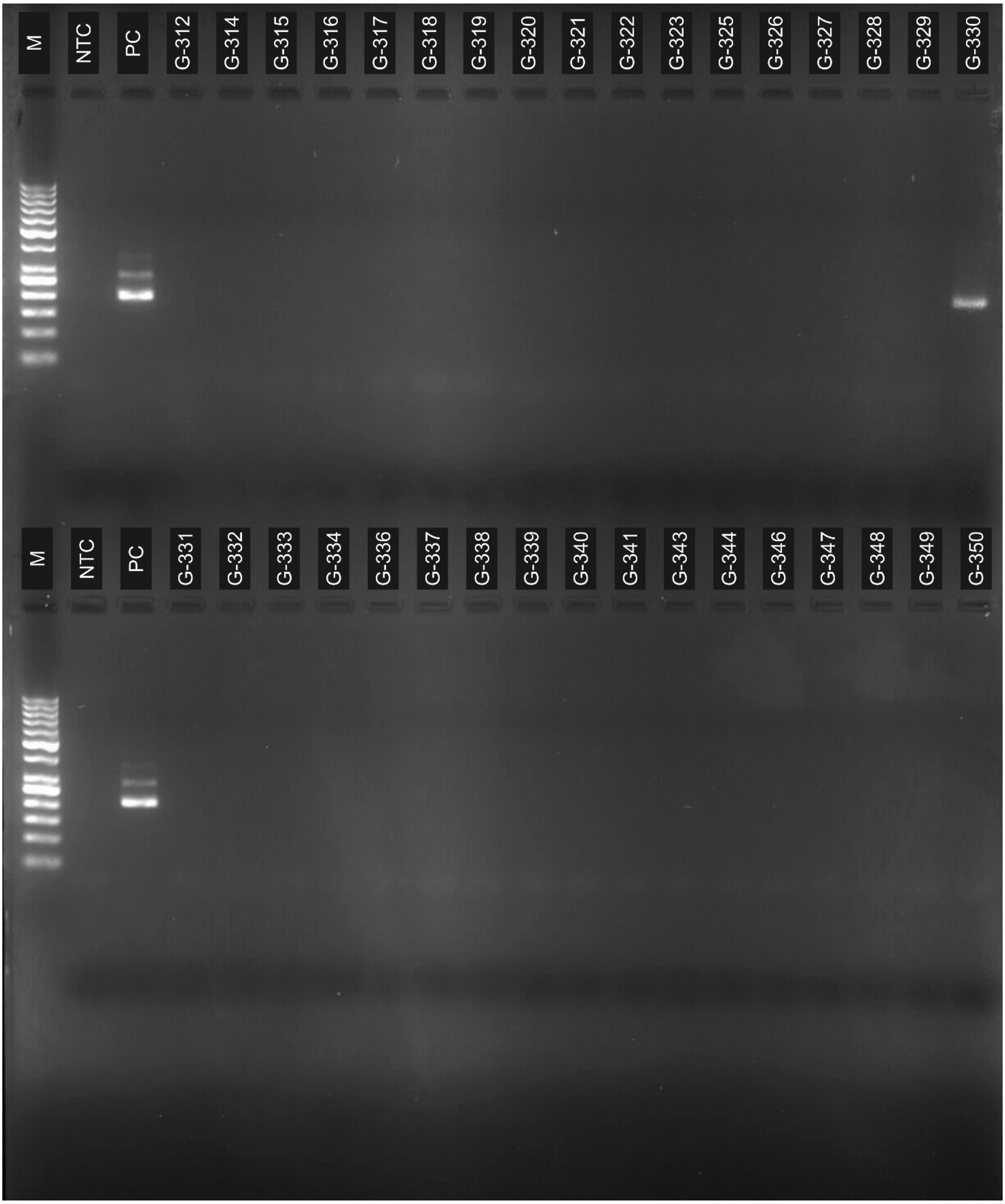
M
NTC
PC
G-312
G-314
G-315
G-316
G-317
G-318
G-319
G-320
G-321
G-322
G-323
G-325
G-326
G-327
G-328
G-329
G-330
M
NTC
PC
G-331
G-332
G-333
G-334
G-336
G-337
G-338
G-339
G-340
G-341
G-343
G-344
G-346
G-347
G-348
G-349
G-350

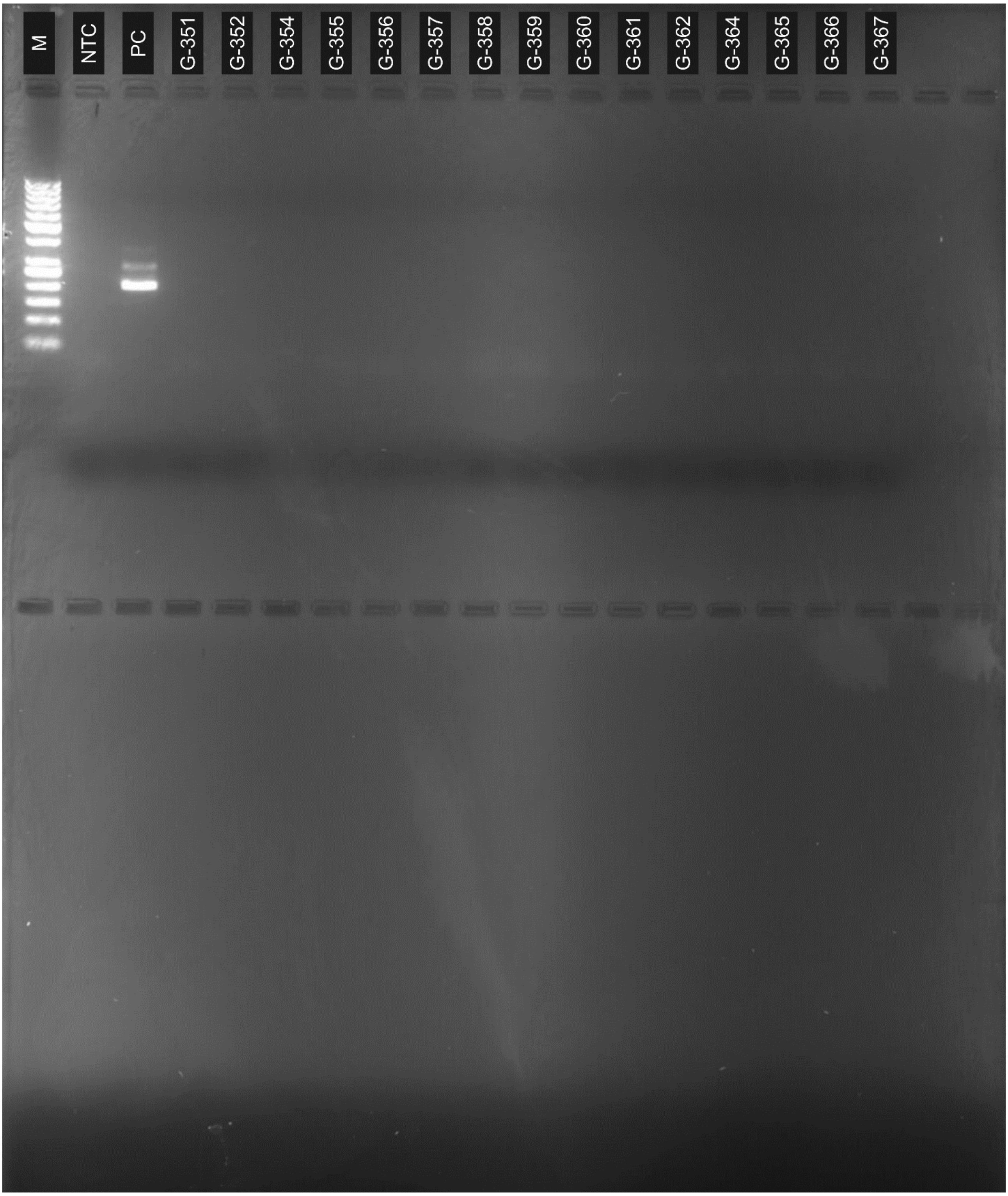
M
NTC
PC
G-351
G-352
G-354
G-355
G-356
G-357
G-358
G-359
G-360
G-361
G-362
G-364
G-365
G-366
G-367

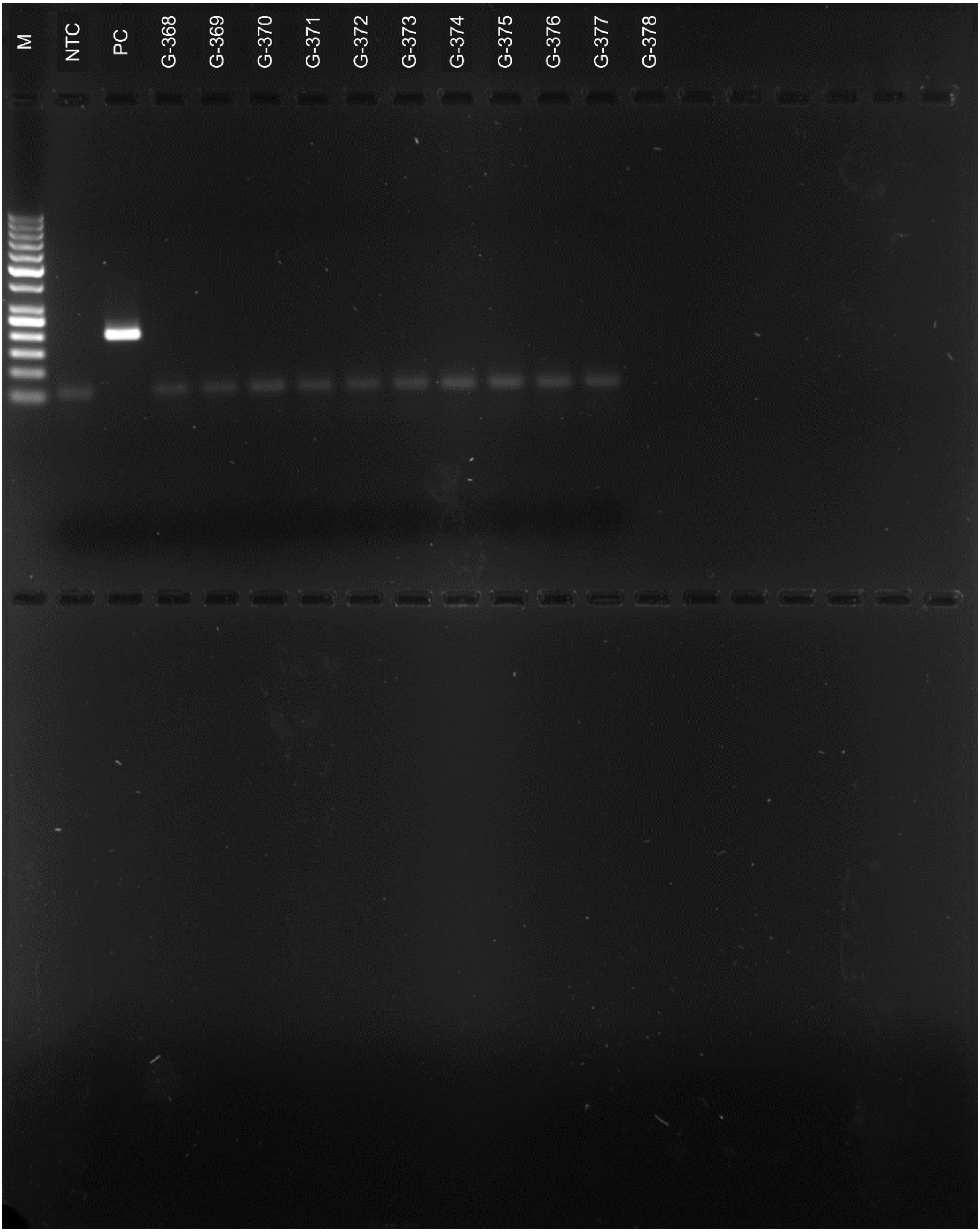
M
NTC
PC
G-368
G-369
G-370
G-371
G-372
G-373
G-374
G-375
G-376
G-377
G-378

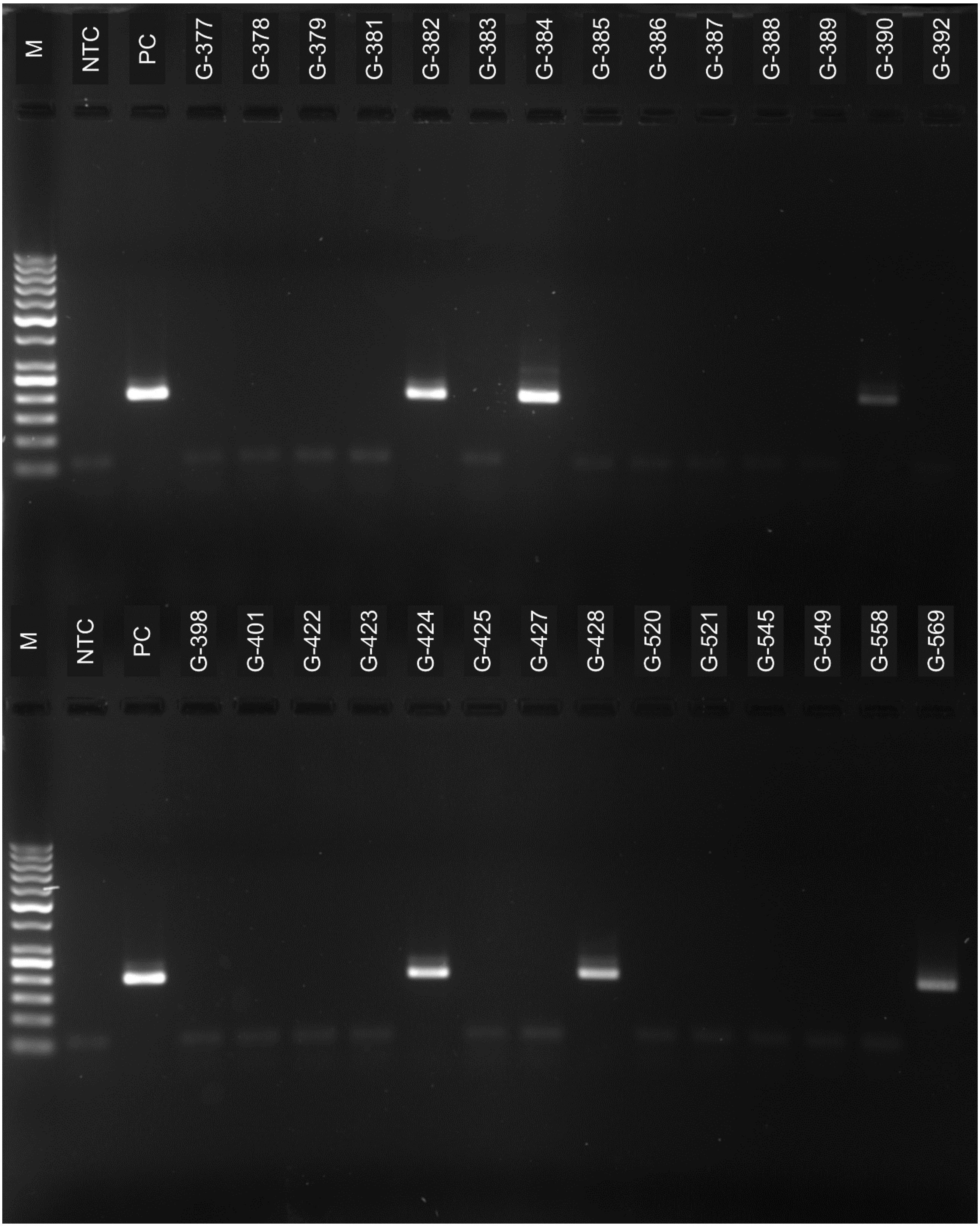
M
NTC
PC
G-377
G-378
G-379
G-381
G-382
G-383
G-384
G-385
G-386
G-387
G-388
G-389
G-390
G-392
M
NTC
PC
G-398
G-401
G-422
G-423
G-424
G-425
G-427
G-428
G-520
G-521
G-545
G-549
G-558
G-569

GÓROWO IŁAWECKIE, BARTOSZYCE DISTRICT, WARMIŃSKO-MAZURSKIE VOIVODSHIP

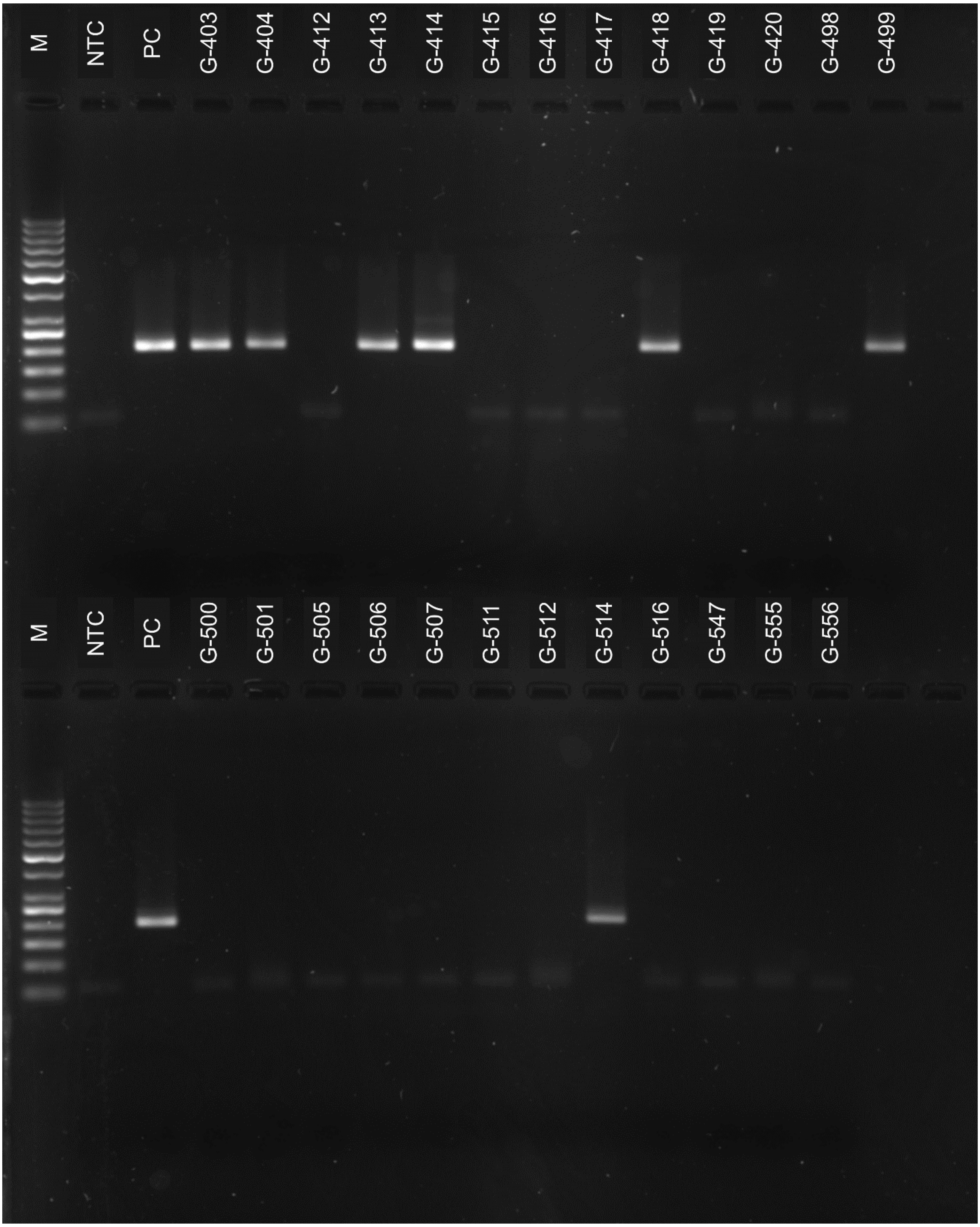

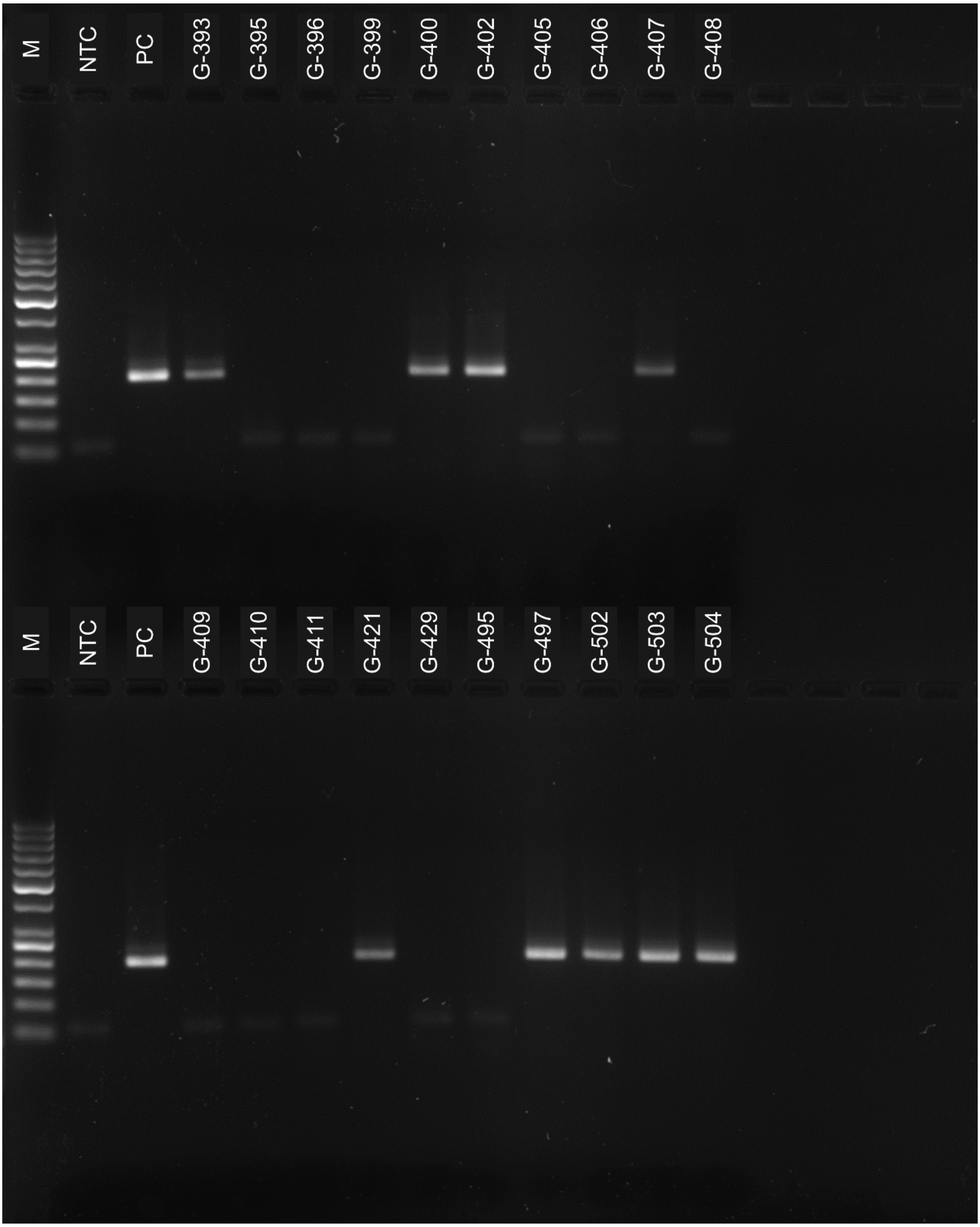
M
NTC
PC
G-393
G-395
G-396
G-399
G-400
G-402
G-405
G-406
G-407
G-408
M
NTC
PC
G-409
G-410
G-411
G-421
G-429
G-495
G-497
G-502
G-503
G-504

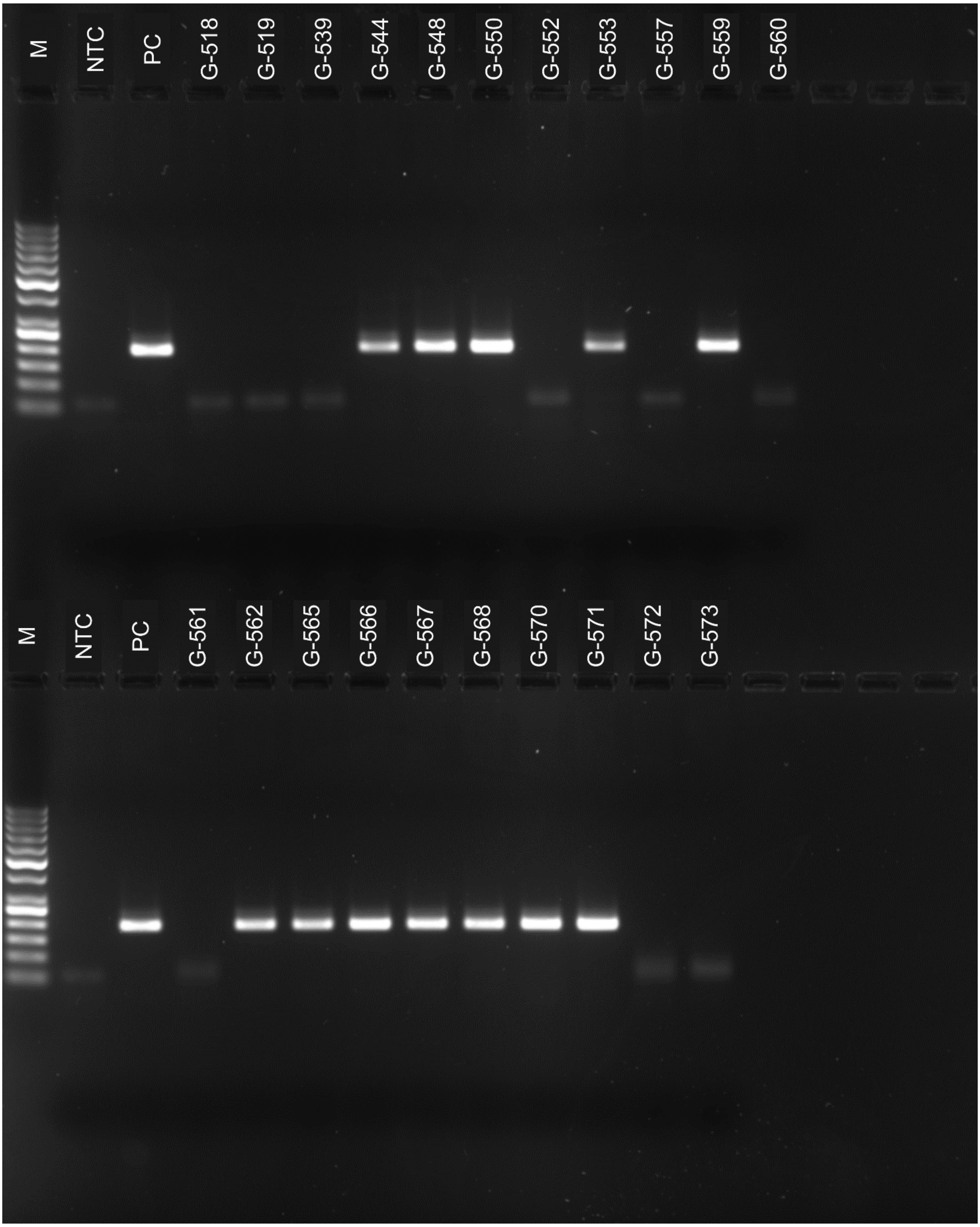
M
NTC
PC
G-518
G-519
G-539
G-544
G-548
G-550
G-552
G-553
G-557
G-559
G-560
M
NTC
PC
G-561
G-562
G-565
G-566
G-567
G-568
G-570
G-571
G-572
G-573

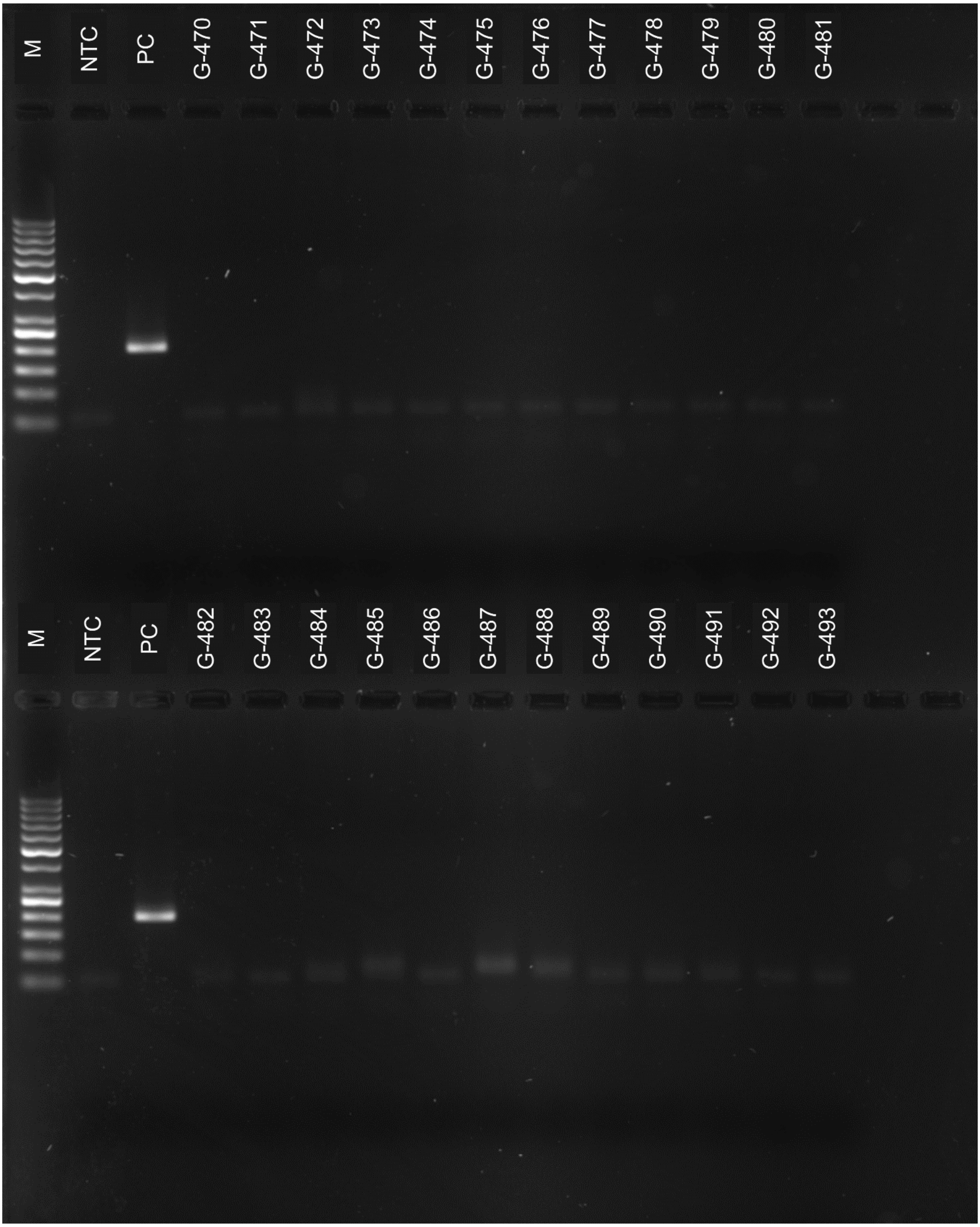
M
NTC
PC
G-470
G-471
G-472
G-473
G-474
G-475
G-476
G-477
G-478
G-479
G-480
G-481
M
NTC
PC
G-482
G-483
G-484
G-485
G-486
G-487
G-488
G-489
G-490
G-491
G-492
G-493

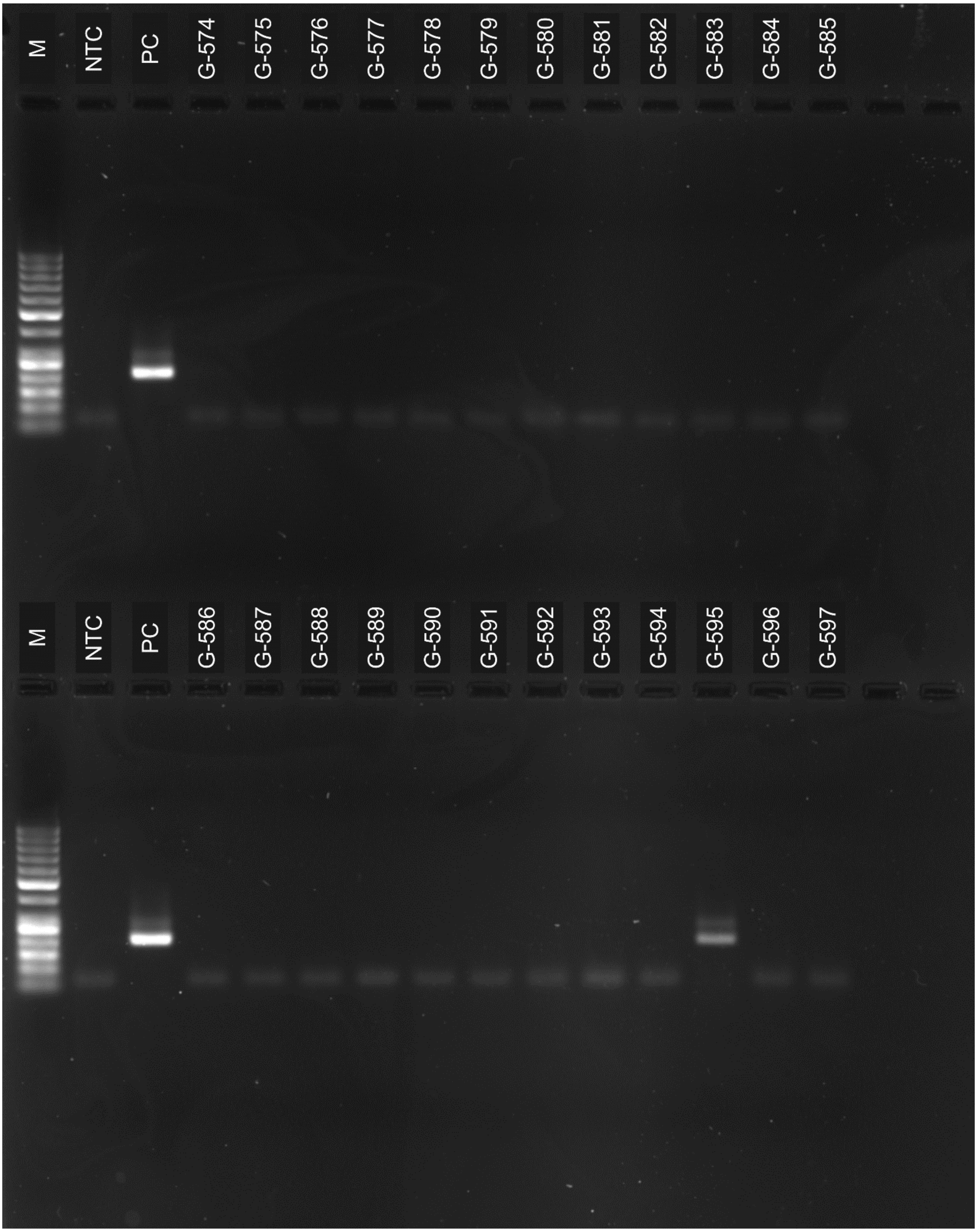
M
NTC
PC
G-574
G-575
G-576
G-577
G-578
G-579
G-580
G-581
G-582
G-583
G-584
G-585
M
NTC
PC
G-586
G-587
G-588
G-589
G-590
G-591
G-592
G-593
G-594
G-595
G-596
G-597

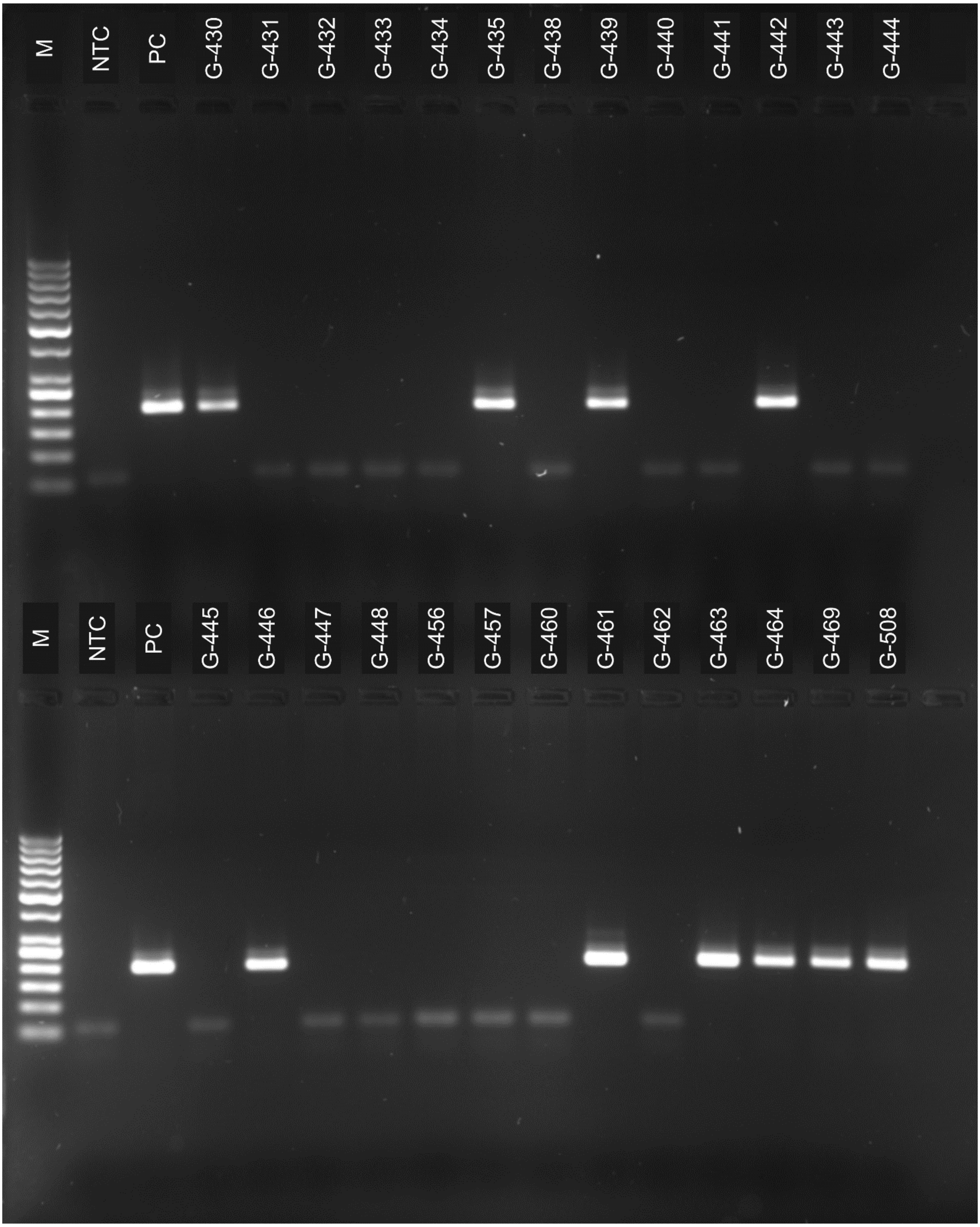
M
NTC
PC
G-430
G-431
G-432
G-433
G-434
G-435
G-438
G-439
G-440
G-441
G-442
G-443
G-444
M
NTC
PC
G-445
G-446
G-447
G-448
G-456
G-457
G-460
G-461
G-462
G-463
G-464
G-469
G-508

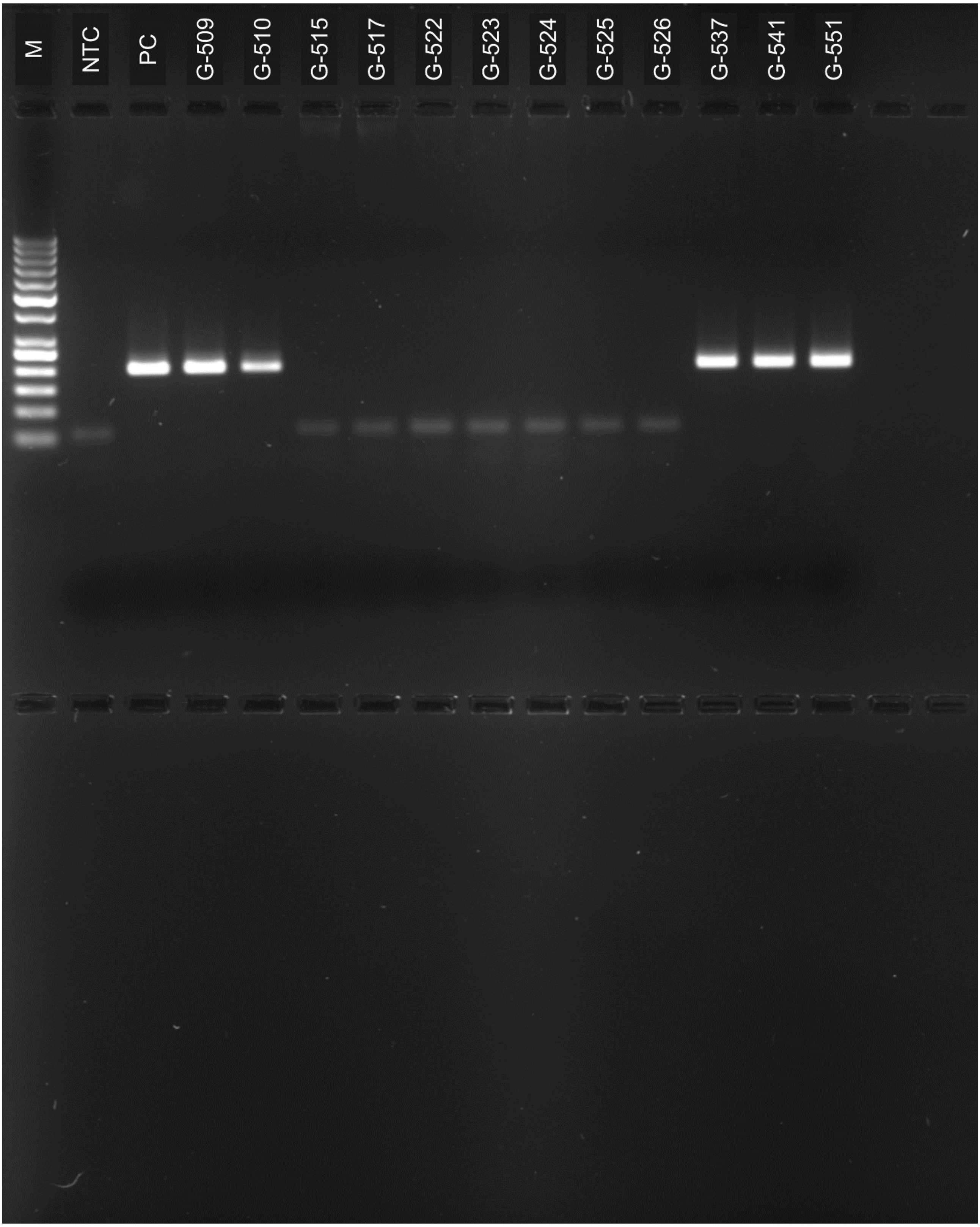
M
NTC
PC
G-509
G-510
G-515
G-517
G-522
G-523
G-524
G-525
G-526
G-537
G-541
G-551

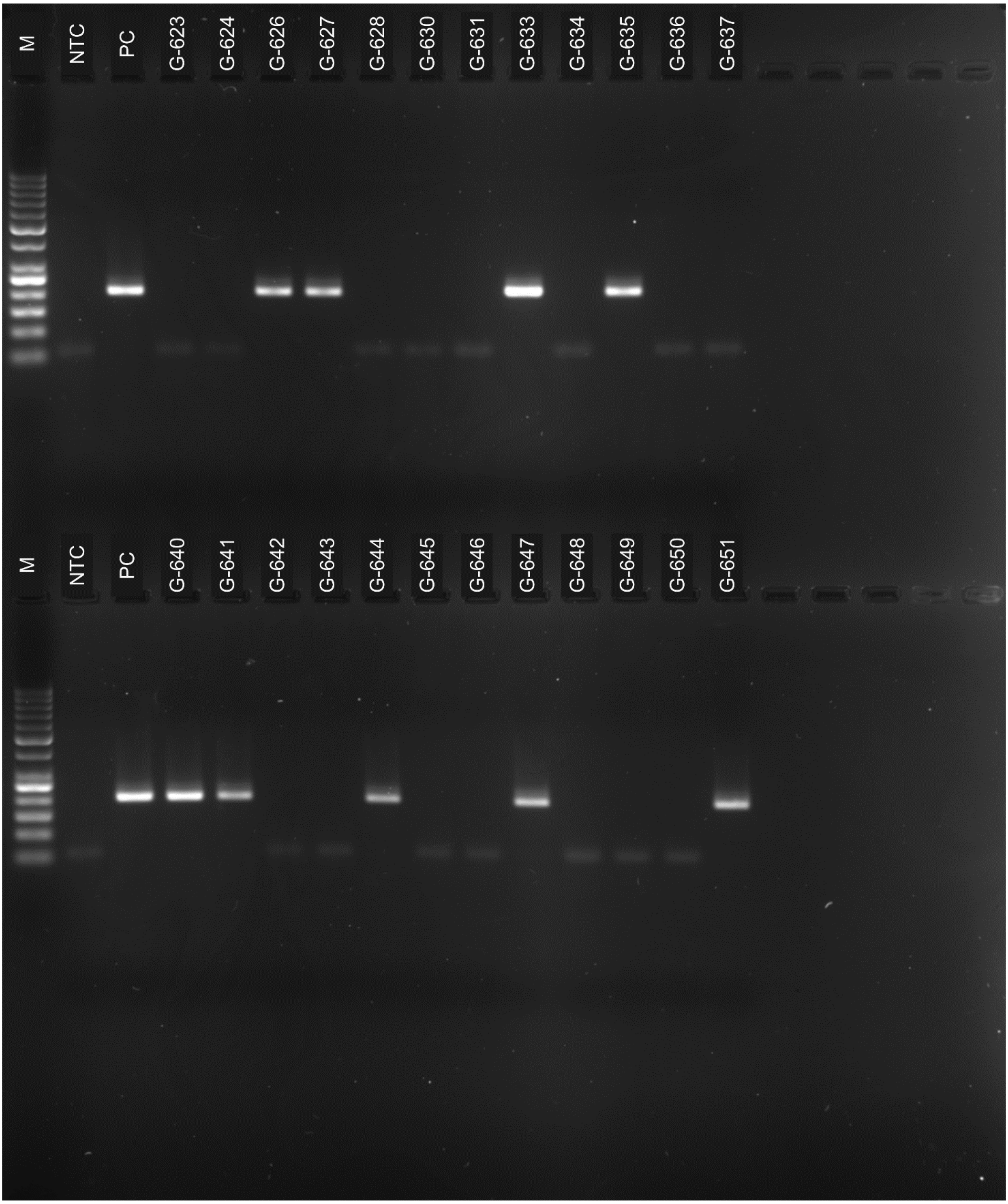
M
NTC
PC
G-623
G-624
G-626
G-627
G-628
G-630
G-631
G-633
G-634
G-635
G-636
G-637
M
NTC
PC
G-640
G-641
G-642
G-643
G-644
G-645
G-646
G-647
G-648
G-649
G-650
G-651
