# Supplementary material for: An update on the prevalence of Echinococcus multilocularis in red foxes (Vulpes vulpes) in a Central European focus: Northern and northeastern Poland (2022–2024)
Source: One Health. 2025 Jul 25;21:101151. doi: 10.1016/j.onehlt.2025.101151 (PMC12329506; doi:10.1016/j.onehlt.2025.101151)
Supplement: Supplementary File 3 — Inhibition control: PCR products visualised on 2 % agarose gels. [file mmc4.pdf]

INHIBITION CONTROL

PCR products visualised on 2% agarose gels. M – GeneRuler 50 bp DNA Ladder (Thermo Scientific, cat. no. SM0371); NTC – no-template control; CTC – cloned-template control.

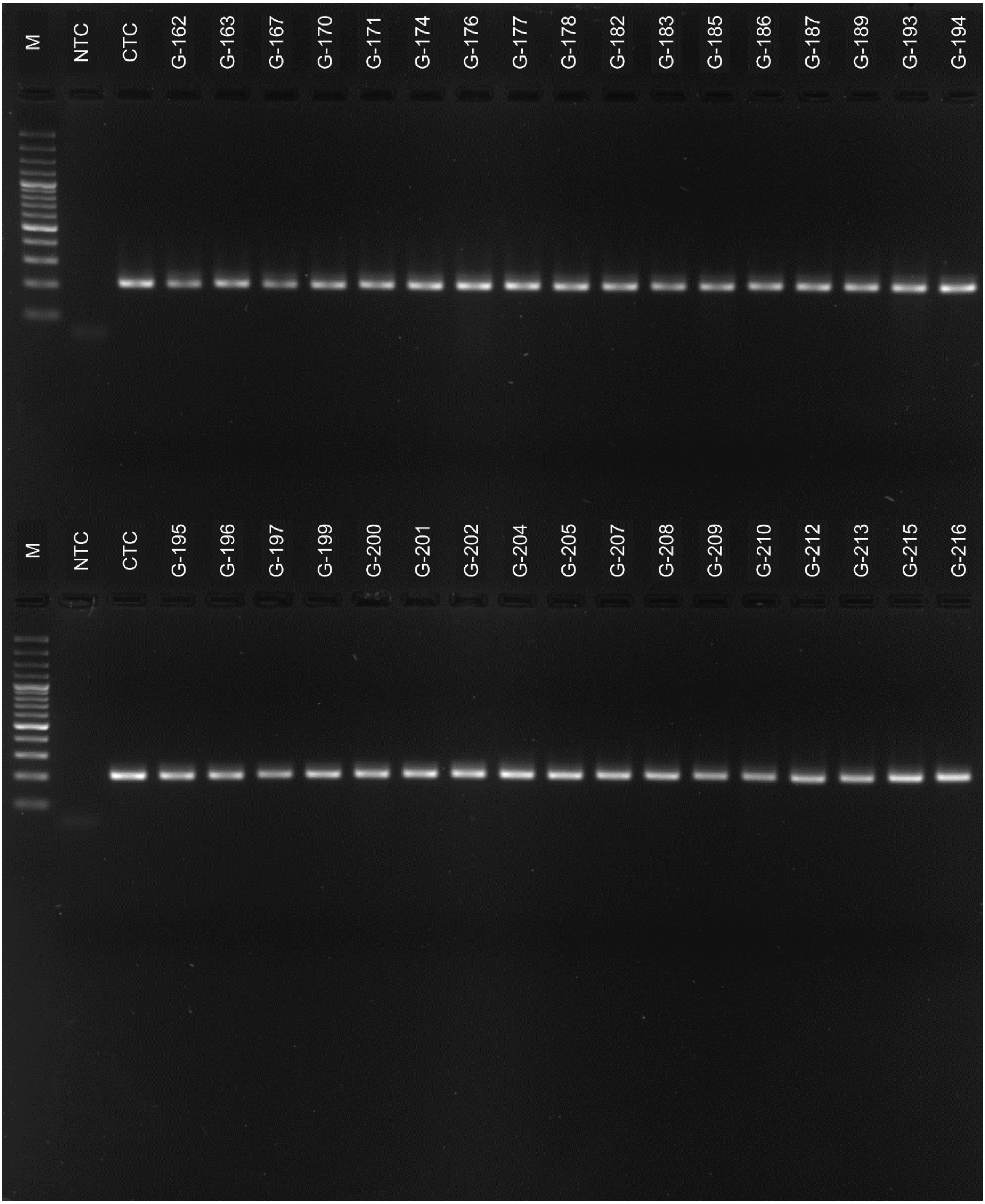

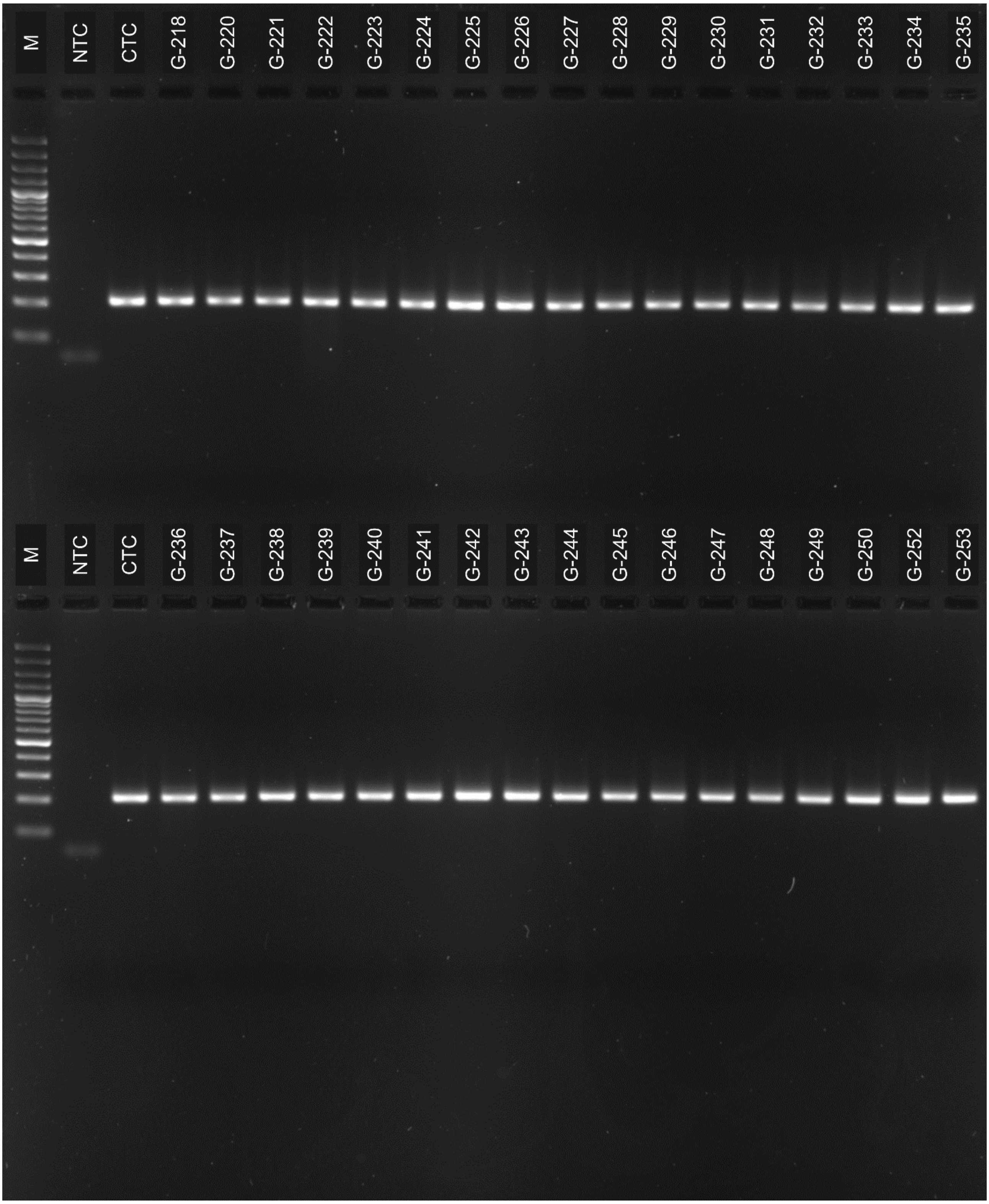
M
NTC
CTC
G-218
G-220
G-221
G-222
G-223
G-224
G-225
G-226
G-227
G-228
G-229
G-230
G-231
G-232
G-233
G-234
G-235
M
NTC
CTC
G-236
G-237
G-238
G-239
G-240
G-241
G-242
G-243
G-244
G-245
G-246
G-247
G-248
G-249
G-250
G-252
G-253

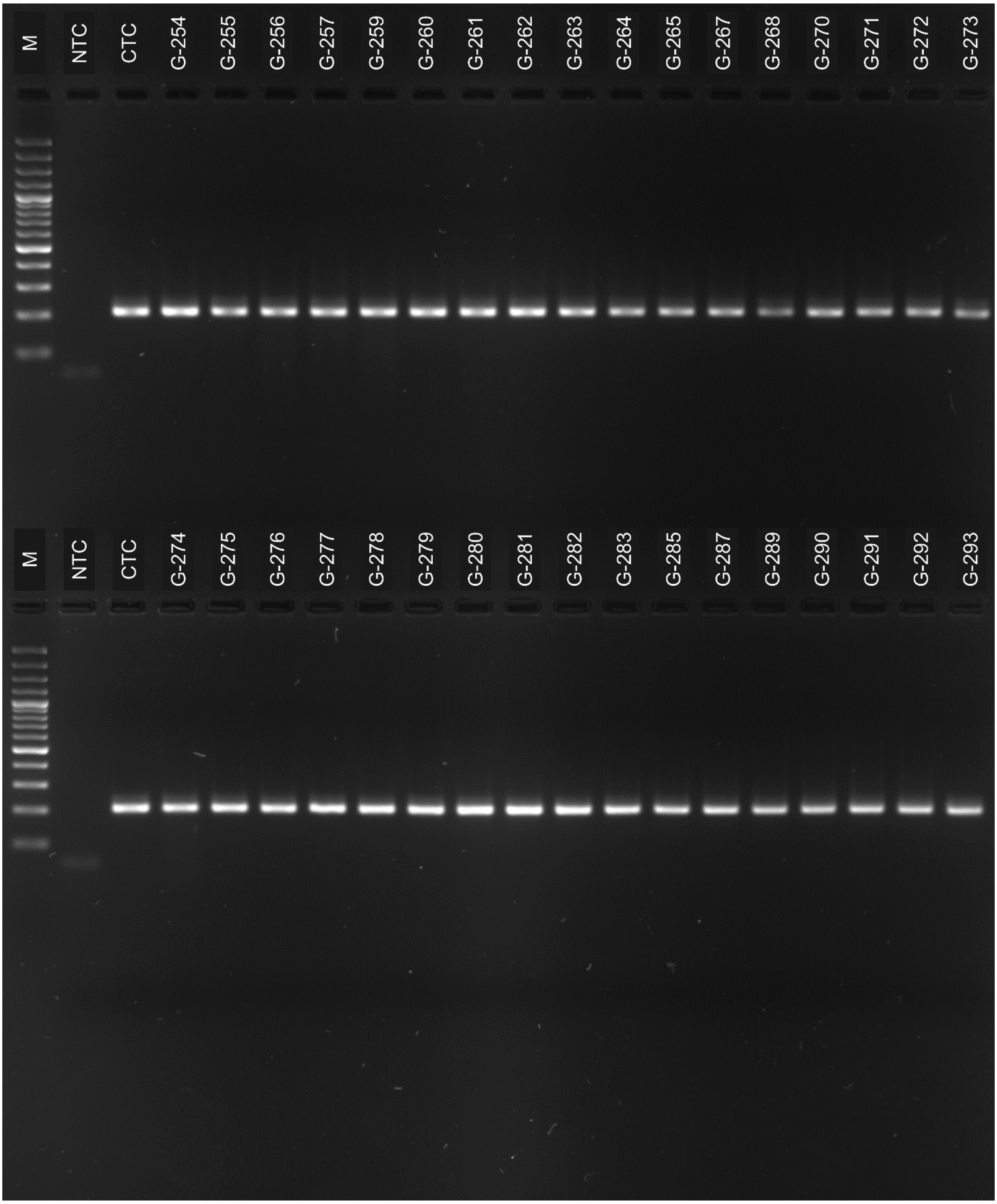
M
NTC
CTC
G-254
G-255
G-256
G-257
G-259
G-260
G-261
G-262
G-263
G-264
G-265
G-267
G-268
G-270
G-271
G-272
G-273
M
NTC
CTC
G-274
G-275
G-276
G-277
G-278
G-279
G-280
G-281
G-282
G-283
G-285
G-287
G-289
G-290
G-291
G-292
G-293

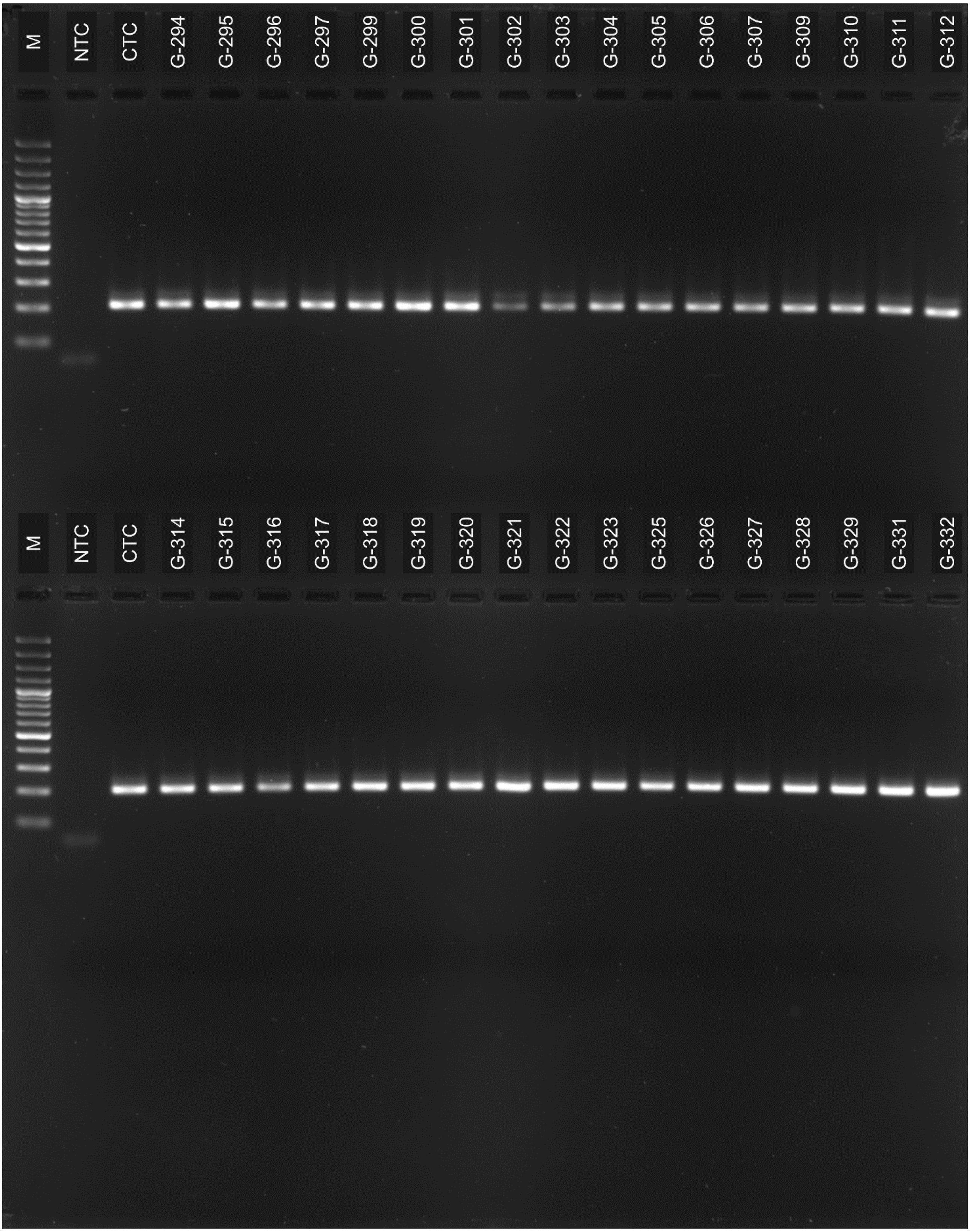
M
NTC
CTC
G-294
G-295
G-296
G-297
G-299
G-300
G-301
G-302
G-303
G-304
G-305
G-306
G-307
G-309
G-310
G-311
G-312
M
NTC
CTC
G-314
G-315
G-316
G-317
G-318
G-319
G-320
G-321
G-322
G-323
G-325
G-326
G-327
G-328
G-329
G-331
G-332

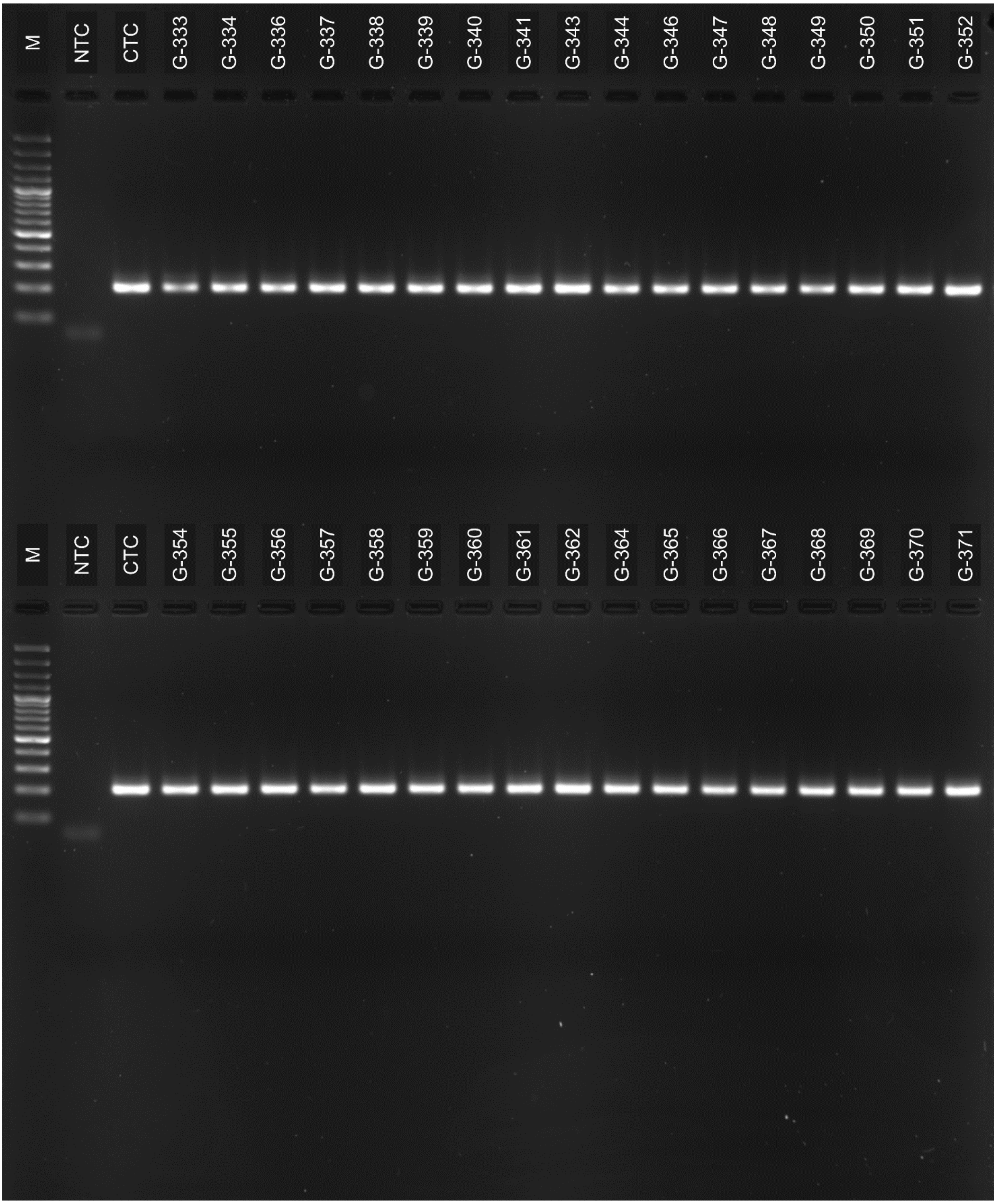
M
NTC
CTC
G-333
G-334
G-336
G-337
G-338
G-339
G-340
G-341
G-343
G-344
G-346
G-347
G-348
G-349
G-350
G-351
G-352
M
NTC
CTC
G-354
G-355
G-356
G-357
G-358
G-359
G-360
G-361
G-362
G-364
G-365
G-366
G-367
G-368
G-369
G-370
G-371

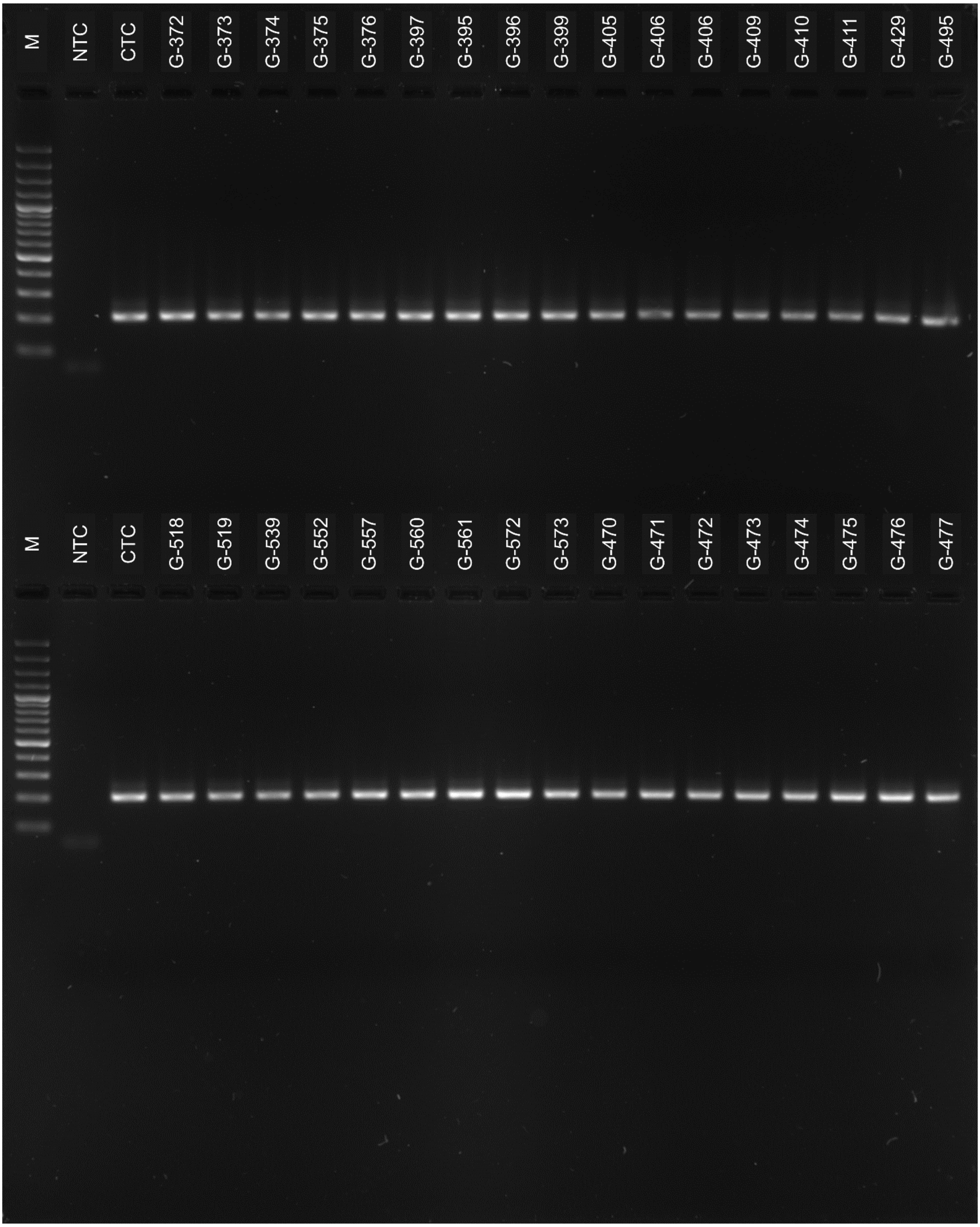
M
NTC
CTC
G-372
G-373
G-374
G-375
G-376
G-397
G-395
G-396
G-399
G-405
G-406
G-406
G-409
G-410
G-411
G-429
G-495
M
NTC
CTC
G-518
G-519
G-539
G-552
G-557
G-560
G-561
G-572
G-573
G-470
G-471
G-472
G-473
G-474
G-475
G-476
G-477

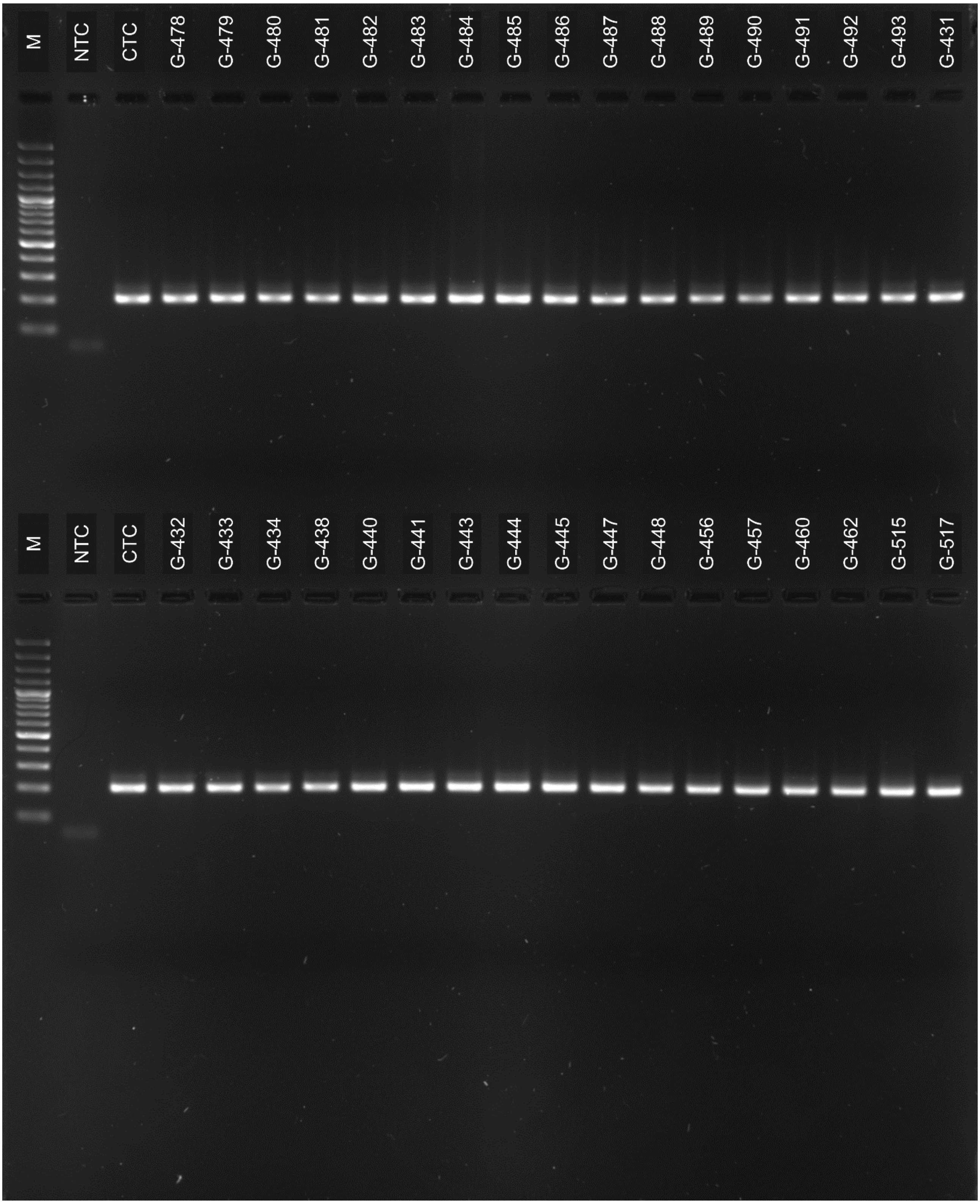
M
NTC
CTC
G-478
G-479
G-480
G-481
G-482
G-483
G-484
G-485
G-486
G-487
G-488
G-489
G-490
G-491
G-492
G-493
G-431
M
NTC
CTC
G-432
G-433
G-434
G-438
G-440
G-441
G-443
G-444
G-445
G-447
G-448
G-456
G-457
G-460
G-462
G-515
G-517

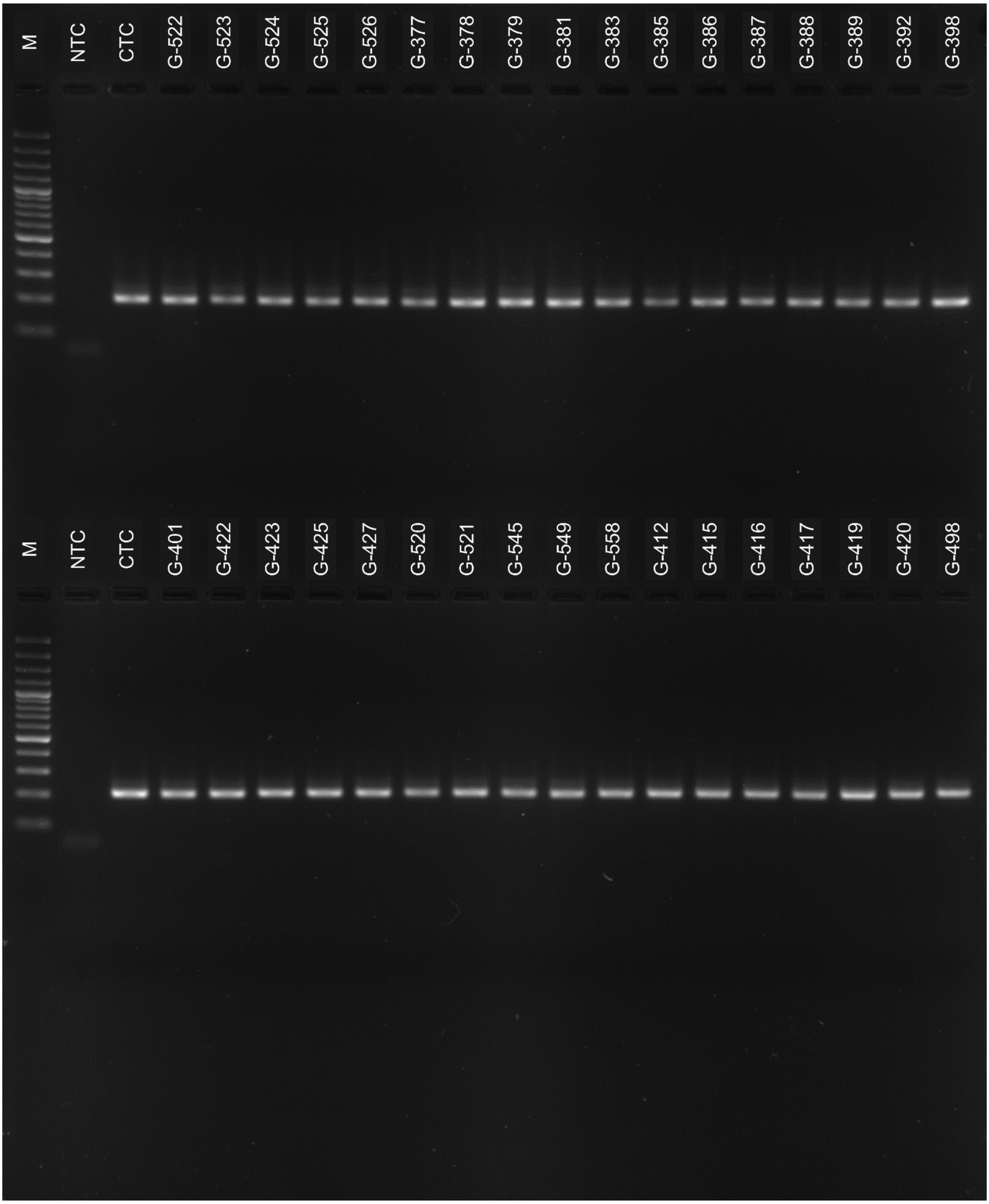
M
NTC
CTC
G-522
G-523
G-524
G-525
G-526
G-377
G-378
G-379
G-381
G-383
G-385
G-386
G-387
G-388
G-389
G-392
G-398
M
NTC
CTC
G-401
G-422
G-423
G-425
G-427
G-520
G-521
G-545
G-549
G-558
G-412
G-415
G-416
G-417
G-419
G-420
G-498

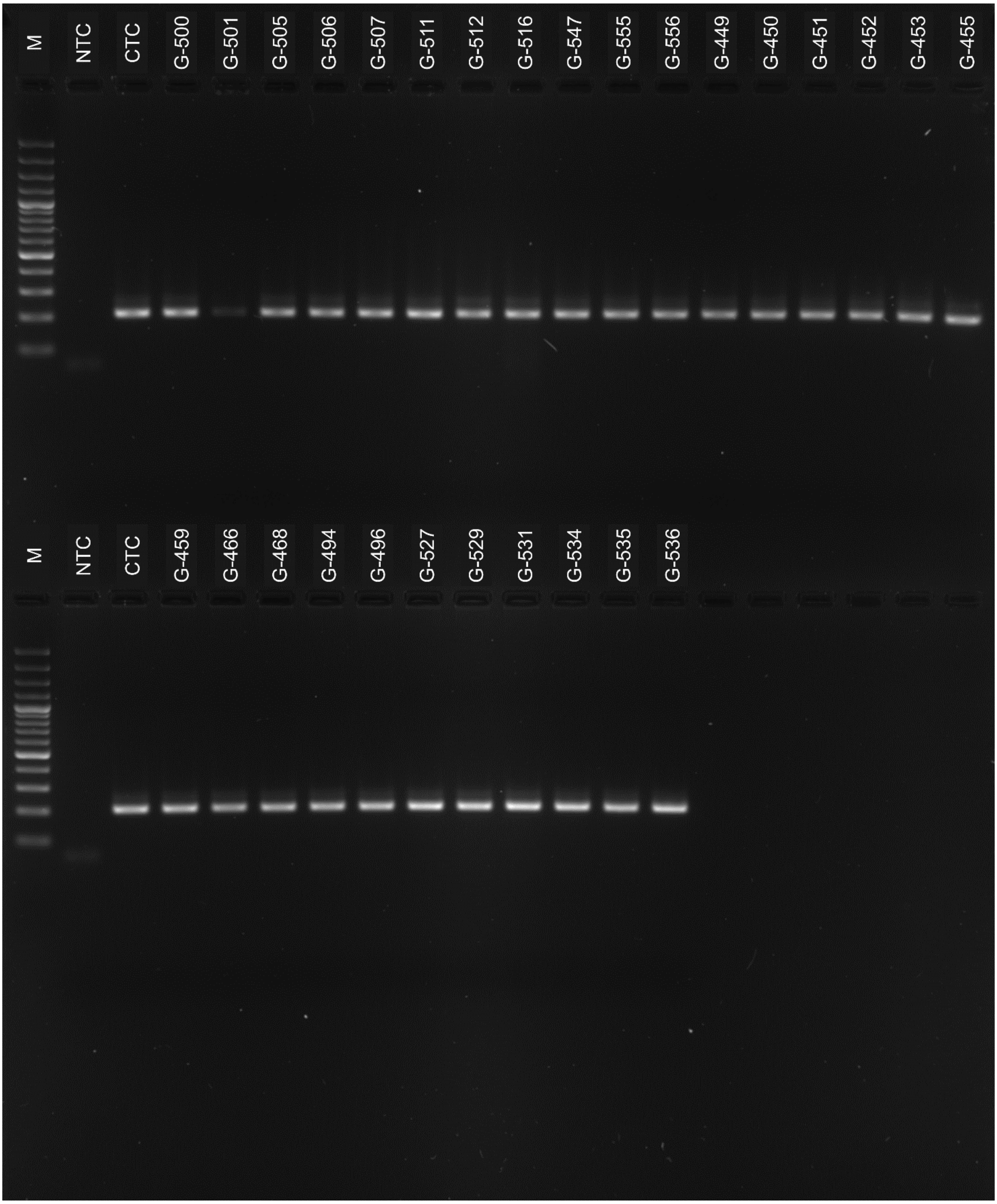
M
NTC
CTC
G-500
G-501
G-505
G-506
G-507
G-511
G-512
G-516
G-547
G-555
G-556
G-449
G-450
G-451
G-452
G-453
G-455
M
NTC
CTC
G-459
G-466
G-468
G-494
G-496
G-527
G-529
G-531
G-534
G-535
G-536

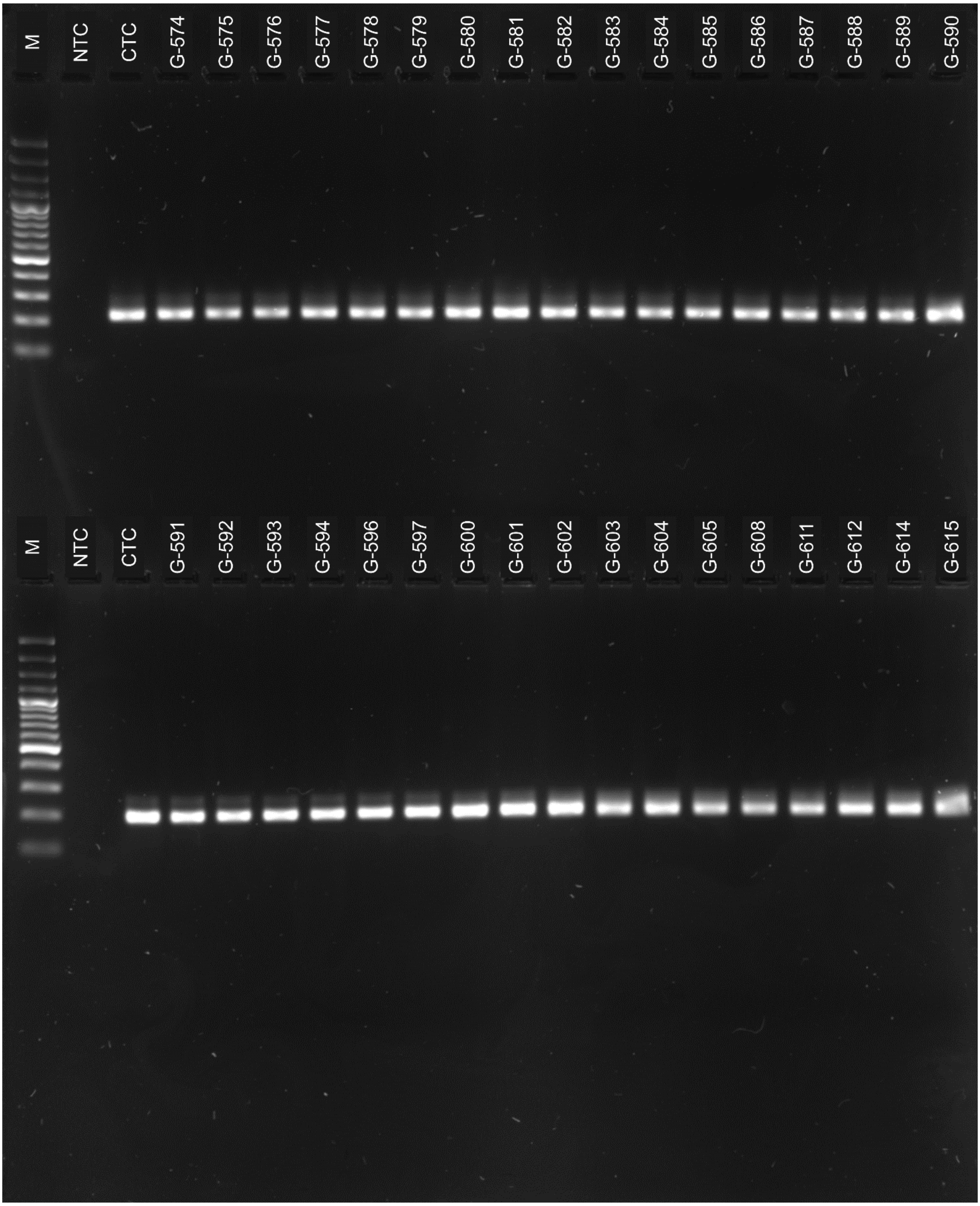
M
NTC
CTC
G-574
G-575
G-576
G-577
G-578
G-579
G-580
G-581
G-582
G-583
G-584
G-585
G-586
G-587
G-588
G-589
G-590
M
NTC
CTC
G-591
G-592
G-593
G-594
G-596
G-597
G-600
G-601
G-602
G-603
G-604
G-605
G-608
G-611
G-612
G-614
G-615

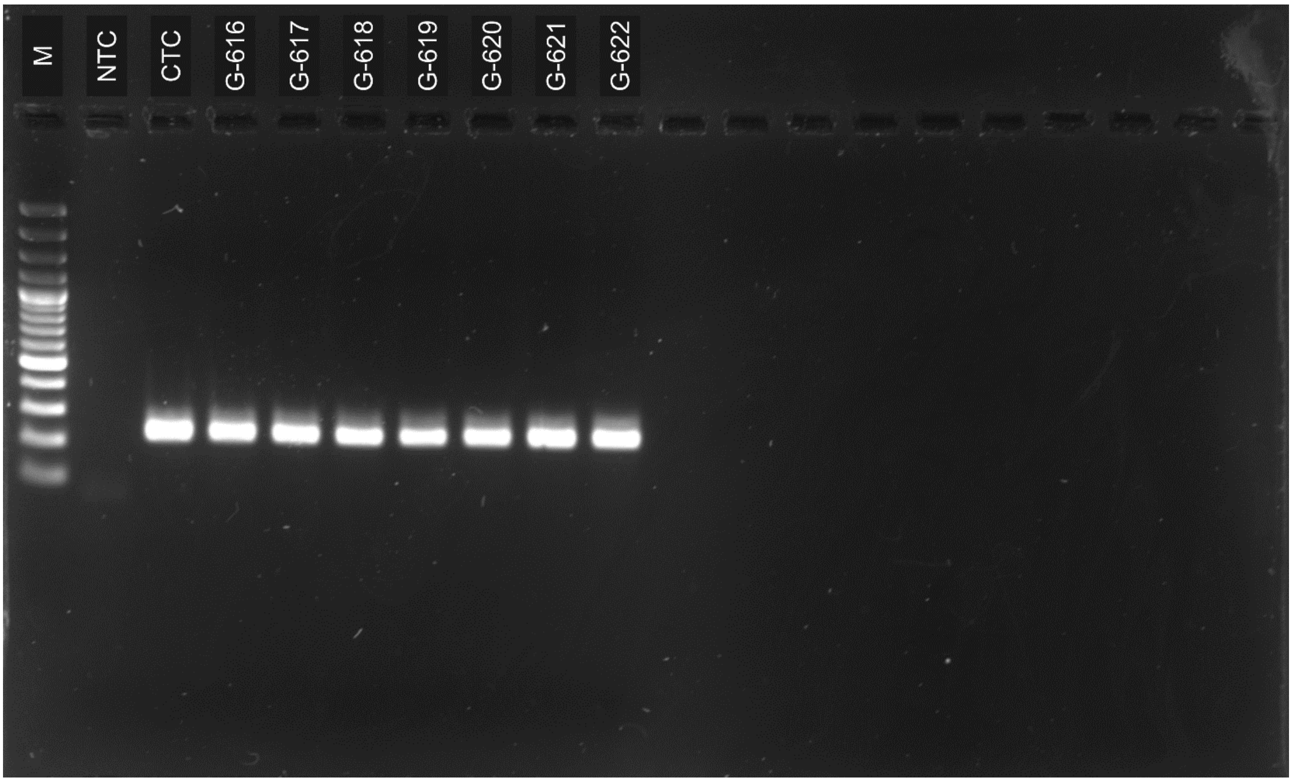
M
NTC
CTC
G-616
G-617
G-618
G-619
G-620
G-621
G-622

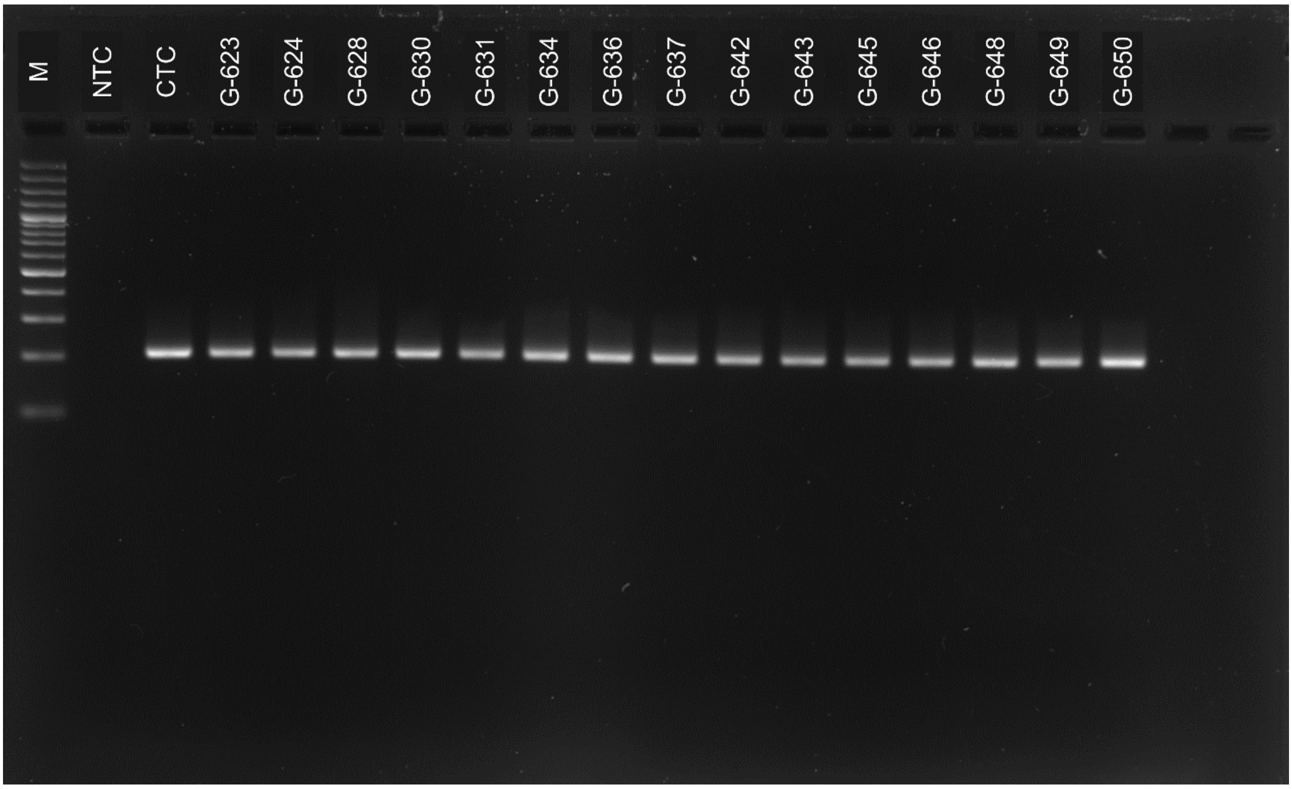
M
NTC
CTC
G-623
G-624
G-628
G-630
G-631
G-634
G-636
G-637
G-642
G-643
G-645
G-646
G-648
G-649
G-650

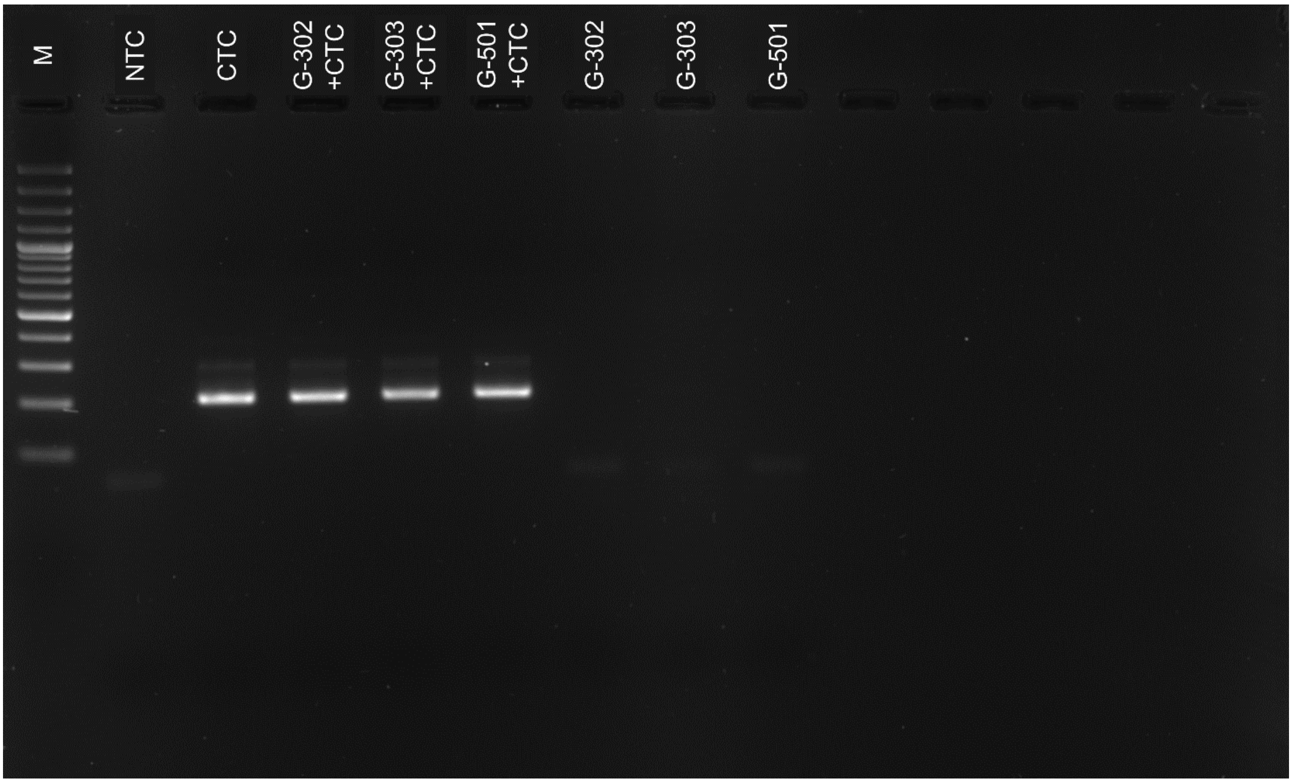
M
NTC
CTC
G-302 +CTC
G-303 +CTC
G-501 +CTC
G-302
G-303
G-501
